# SUMO specific peptidase 3 halts pancreatic ductal adenocarcinoma metastasis via deSUMOylating DKC1

**DOI:** 10.1038/s41418-023-01175-4

**Published:** 2023-05-15

**Authors:** Xiao Wu, Jian-Hui Li, Long Xu, Ya-Xiong Li, Xiao-Xu Zhu, Xi-Yu Wang, Xingmei Wu, Wei Zhao, Xuhao Ni, Xiao-Yu Yin

**Affiliations:** 1 Department of Pancreato-Biliary Surgery, the First Affiliated Hospital of Sun Yat-Sen University, Guangzhou, 510080 China; 2 Department of Physiology, Zhongshan School of Medicine, Sun Yat-Sen University, Guangzhou, 510080 China; 3 Department of Otorhinolaryngology, the First Affiliated Hospital of Sun Yat-Sen University, Guangzhou, 510080 China; 4 Key Laboratory of Stem Cells and Tissue Engineering (Sun Yat-Sen University), Ministry of Education, Guangzhou, 510080 China

**Keywords:** Tumour-suppressor proteins, Sumoylation

## Abstract

In the past few decades, advances in the outcomes of patients suffering from pancreatic ductal adenocarcinoma (PDAC) have lagged behind these gained in the treatment of many other malignancies. Although the pivotal role of the SUMO pathway in PDAC has been illustrated, the underlying molecule drivers have yet to be fully elucidated. In the present study, we identified SENP3 as a potential suppressor of PDAC progression through an in vivo metastatic model. Further studies revealed that SENP3 inhibited PDAC invasion in a SUMO system dependent fashion. Mechanistically, SENP3 interacted with DKC1 and, as such, catalyzed the deSUMOylation of DKC1, which accepted SUMO3 modifiers at three lysine residues. SENP3-mediated deSUMOylation caused DKC1 instability and disruption of the interaction between snoRNP proteins, which contributed to the impaired migration ability of PDAC. Indeed, overexpression of DKC1 abated the anti-metastasis effect of SENP3, and DKC1 was elevated in PDAC specimens and associated with a poor prognosis in PDAC patients. Collectively, our findings shed light on the essential role of SENP3/DKC1 axis in the progression of PDAC.

## Introduction

As the most common pancreatic cancer, pancreatic ductal adenocarcinoma (PDAC) features with poor 5-year overall survival (OS) rate (approximately 9%) and becomes a frequent cause of cancer-related mortality [1]. Surgical resection is still the most effective treatment for PDAC. However, due to the local advance or distant metastasis, only 20% of patients have surgical opportunity when diagnosed [2, 3]. In the past decades, although advances in diagnostic approaches, perioperative management, radiotherapy techniques, and systemic therapies have enriched the arsenal for PDAC treatment, only modest progress has been achieved [4–6]. Therefore, it is imperative to find effective ways to improve the survival of patients with PDAC.

Post-translational modifications (PTMs), including phosphorylation, methylation, ubiquitination, and SUMOylation, play an essential role in modulating maturation, degradation, structural formulation as well as subcellular distribution of proteins, and therefore enrich protein functions and regulatory network [7, 8]. Among those modifications, SUMOylation is a process in which small ubiquitin-like modifiers (SUMOs) are covalently conjugated into lysine residues of target protein containing a consensus motif [9]. Like dynamic ubiquitination, modifier-activating enzyme E1 (Aos1/Uba2), conjugating enzyme E2 (UBC9) and substrate-specific ligase E3 are involved in this cascade reaction, which can be reversed by a family of sentrin/SUMO-specific proteases (SENPs) [10–12]. In the last decades, the role of SUMO pathway in mediating tumor progression has been widely reported [13, 14]. In PDAC, SUMO pathway was reported to be associated with MYC hyperactivation and poor prognosis in patients [15]. Additionally, the therapeutic potential of a novel SEA-targeting SUMO inhibitor was investigated in PDAC and the results showed that the anti-tumor immunity was provoked by abating SUMO pathway [16]. However, the detailed mechanisms underlying the SUMO-mediated pro-tumor effect remain poorly understood.

The dyskerin pseudouridine synthase (DKC1) gene was first identified in dyskeratosis congenita (DC) [17]. DKC1 is one of the core proteins in box H/ACA snoRNPs, which consist of three other highly conserved proteins (NOP10, NHP2 and GAR1) and play an indispensable role in maintaining the stability and activity of telomerase [18, 19]. In addition, emerging evidence suggested that DKC1 was involved in regulating other cellular processes, such as IRES-mediated translation [20] and post-transcriptional processing of precursor RNA [21]. Recently, many studies demonstrated that dysregulation of DKC1 was associated with tumor growth and metastasis as well as prognosis of patients suffering from various human cancer types, including brain [22], lung [23], breast [24], liver [25] and colon [26].

In the present study, we identified SENP3 as a metastatic inhibitor in PDAC using an in vivo metastatic model screening SUMO-specific proteases. In addition, the expression of SENP3 was reduced in PDAC tissues and correlated with a favorable prognosis in patients. Mechanistically, SENP3 was proven to interact with DKC1 and therefore catalyze the deSUMOylation of DKC1 at K413, K448 and K467. DeSUMOylated DKC1 was instable and partly lost its association with NHP2, which was mediated by SUMO interaction motifs (SIMs). Further studies unveiled that overexpression of DKC1 could undermine the anti-metastasis effect of SENP3, and a high level of DKC1 predicted poorer outcomes in PDAC patients.

## Results

### SENP3 expression is reduced in PDAC and associated with a favorable prognosis

**Fig. 1:**
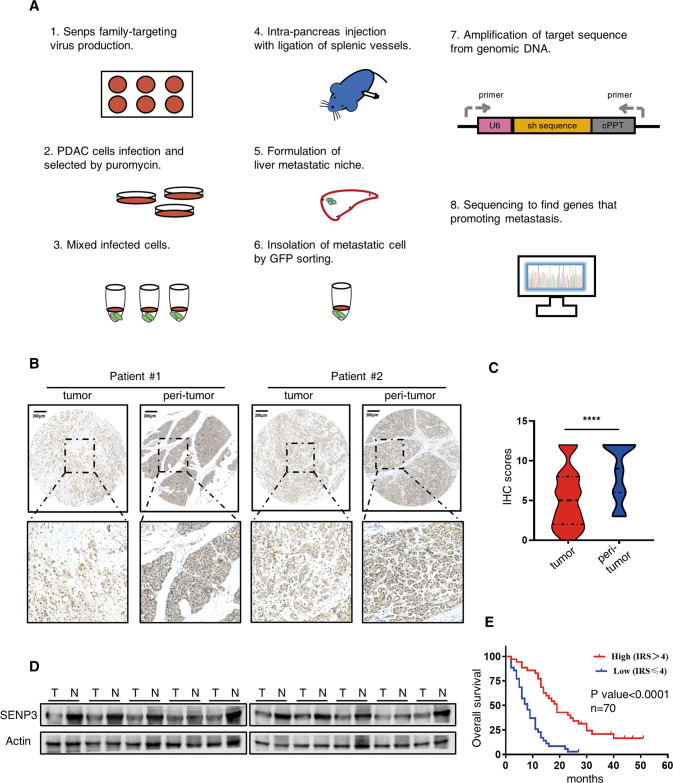
SENP3 is downregulated in PDAC tissues and correlated with a favorable prognosis. **A** Schema of an in vivo screening strategy for identifying SENPs that modulate PDAC metastasis. Lentivirus particles targeting each SENP family member were individually produced in HEK293T cells and subsequently infected into GFP and luciferase-producing PDAC cell lines (PANC-1 and Patu-8988t). Infected cells were selected with puromycin and then mixed (for each SENP-targeted cell line 3 × 10^4^ cells were employed) for orthotopic injection into pancreas of ten nude mice for each cell line. Eight weeks later, vigorous metastatic liver nodules detected with the IVIS imaging system were isolated from tumor-bearing mice. GFP-positive metastatic cells were isolated with FACS sorting following single-cell suspension collection by liver digestion. Genomic DNA extracted from sorted cells was amplified and verified by DNA sequencing. **B** Representative IHC image of SENP3 in PDAC and paired peri-tumor tissues using PDAC tissue microarray(TMA), Scale bars shown is 200 μm. **C** Statistical analysis of IHC scores from TMA was performed, unpaired *t* test. ****p < 0.0001. **D** Expression of SENP3 was determined by western blot analysis in ten paired primary pancreatic cancer tissues (T) and the matched adjacent nontumor tissues (N) from our center. **E** Overall survival based on SENP3 expression in TAM. log-rank test.

Although the essential role of SUMO pathway in pancreatic ductal adenocarcinoma (PDAC) was demonstrated in several literatures [15, 16, 27], the molecular drivers of SUMO-mediated regulation remain not fully understood. We performed a shRNA screening to identify candidates that could modulate the metastasis of PDAC (Fig. 1A). For this, we produced a set of shRNA lentiviruses targeting seven SUMO specific peptidases (SENPs) and individually introduced them into two PDAC cell lines (Patu-8988t and PANC-1). After puromycin selection and verification of knockdown efficiency for each gene (Fig. S1A), an equal number of cells from each shRNA clone (3 × 10^4^ per clone) was mixed for orthotopic injection into nude mice pancreas. After eight weeks following inoculation, seven of ten mice bearing PANC-1 cells and nine of ten mice challenged with Patu-8988t cells were detected with liver metastatic signals using the IVIS imaging system. The five most obvious metastatic nodules for each cell line were isolated and subjected to genomic DNA extraction. Subsequent sequencing results revealed that 3 of 5 obtained nodules from PANC-1-bearing mice contained sequences targeting SENP3 (1 for SENP5 and 1 for SENP6), whereas all metastatic tumors in mice inoculated with Patu-8988t cells expressed SENP3-targeting sequences (Fig. S1C), indicating that knockdown of senp3 promoted PDAC metastasis in vivo. To further confirm the clinical importance of our findings, the prognostic influence of each SENP was analyzed using the online GEPIA2 datasets, and the results showed that only senp3 was correlated with the outcome of PDAC patients in a favorable manner (Fig. S1D). Further analysis revealed that these favorable outcomes might be ascribed to the lower burden of gene mutations (such as KRAS and TP53) in the patients with higher senp3 expression (Fig. S1E, F).

Next, we sought to determine the SENP3 protein expression in human PDAC tissue microarray (TMA), and the immunohistochemistry (IHC) results showed that SENP3 was downregulated in PDAC specimens compared with normal tissues (Fig. 1B, C). Consistently, we also determined the SENP3 levels in PDAC and paired adjacent nontumor tissues in our center by immunoblot and found that the expression of SENP3 was evidently lower in PDAC (Fig. 1D). Moreover, patients with higher IHC scores showed a favorable prognosis in comparison with their counterparts (Fig. 1E). Collectively, SENP3 may play a protective role among patients with PDAC.

### SENP3 shows a negative effect on PDAC metastasis

**Fig. 2:**
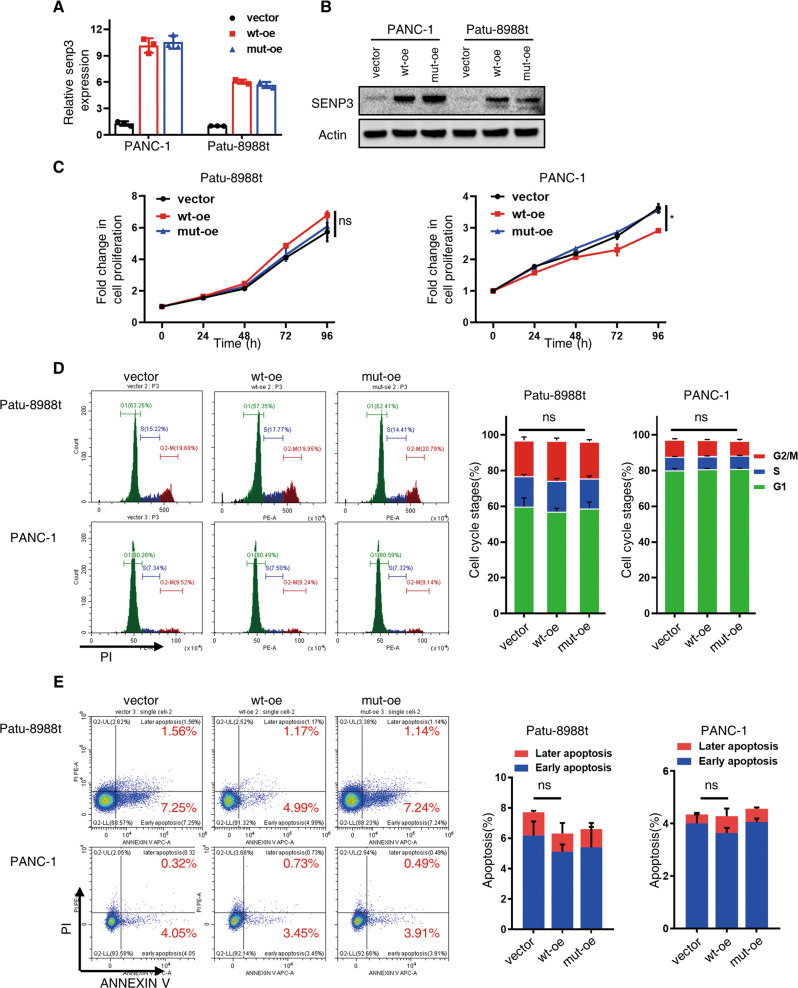
SENP3 is dispensable for PDAC proliferation. SENP3 expression in cells was determined by RT-qPCR (**A**) and western blot (**B**). **C** Cell growth curve of Patu-8988t and PANC-1 cells endogenous overexpressing of vector or wild-type SENP3 or inactive mutant SENP3 via CCK8 assay. **D** Cell cycle analysis of Paut-8988t and PANC-1 cells endogenous overexpressing of vector or wild-type SENP3 or inactive mutant SENP3. **E** Apoptosis analysis of Paut-8988t and PANC-1 cells endogenous overexpressing of vector or wild-type SENP3 or inactive mutant SENP3. Unpaired *t* test, ns, no significance; *p < 0.05.

**Fig. 3:**
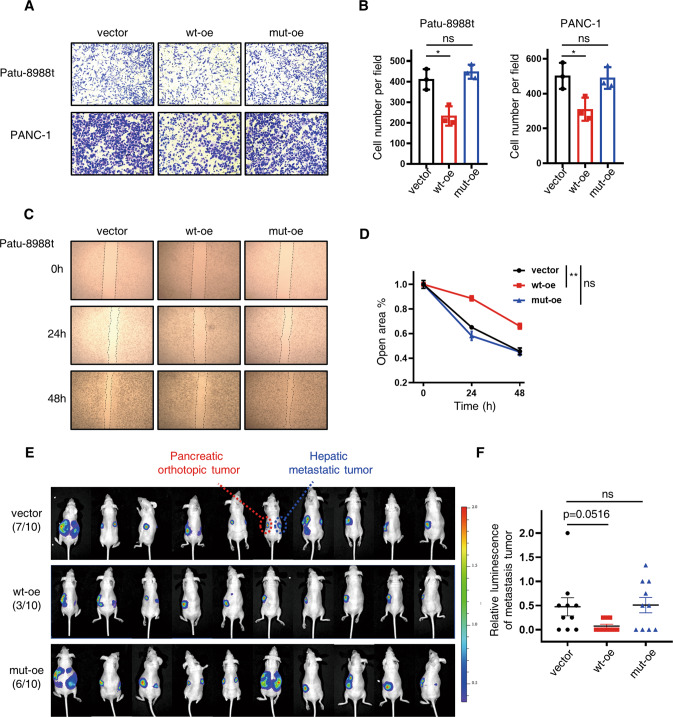
Wild-type SENP3 inhibits PDAC metastasis. **A**, **B** Transwell assay detected the migration ability after endogenous overexpression of wild-type SENP3 (wt-oe) or enzymatically defective SENP3 mutants (C532S, mut-oe) in Patu-8988t cells and PANC-1 cells. **C**, **D** Wound healing assay analyzed the migration ability of Patu-8988t cells infected with indicated overexpression vectors. Statistical analysis was performed according to scratch width at 48 h. **E**, **F** The in vivo migration potency of vector control, wild-type SENP3, C532S SENP3-expressing Patu-8988t cells. Luciferase-expressing Patu-8988t cells were subjected to lentiviral infection to express either vector control (vector), wild-type SENP3 (wt-oe) or catalytically deficient C532S SENP3 (mut-oe) and then injected into pancreases of mice, luciferase signal was monitored weekly, images in **E** was detected at eight weeks after inoculation. Relative luciferase signal of metastatic signal (**F**) was calculated using relative metastatic signal/ orthotopic signal. Unpaired *t* test, no significance, *p < 0.05, **p < 0.01.

To unveil the role of SENP3 and its enzymatic activity in the progression of PDAC, we established stable wild-type SENP3 over-expressing (wt-oe) PDAC cells, as control empty vector and catalytically inactive SENP3 (C532S, mut-oe) was also expressed in these cells via lentiviral infection (Fig. 2A, B). Although another SUMO-specific protease, SENP1, was demonstrated to promote PDAC cell growth [28], our in vitro proliferation assays, including CCK8 tests (Fig. 2C), cellular cycle assays (Fig. 2D), indicated that SENP3 was dispensable for PDAC growth. Moreover, there were no evident differences in the percentages of apoptotic cells among cells with different expression of SENP3 (Fig. 2E). On the other hand, elevated expression of functionally competent SENP3 reduced the cellular migration as detected by transwell assays, whereas this effect was diminished in cells expressing mutated SENP3 (Fig. 3A, B). Consistently, similar results were shown in the wound healing assays (Figs. 3C, D, S2A).

To further determine the anti-metastasis effect of SENP3 on PDAC in vivo, we orthotopically injected PDAC cells (Patu-8988t cells endogenously expressing empty vector or wild-type SENP3 or mutated SENP3) into the pancreas of nude mice. Eight weeks later, fluorescence imaging showed that the metastasis rate of mice challenged with cell expressing wild-type SENP3 (3/10) was obviously lower than the counterparts (7/10 in the vector group, 6/10 in the mut-oe group) (Fig. 3E). Further analysis revealed that the relative intensity of metastatic foci in the wt-oe group was also lower than other two groups (Fig. 3F). In addition, endogenous over-expression of SENP3 by dCas9-CRISPR system consistently reduced the migration ability of PDAC cells (Fig. S3). In summary, SENP3 inhibits PDAC metastasis both in vitro and in vivo, which depends on its enzymatic potency in the SUMO system.

### SENP3 colocalizes with DKC1

**Fig. 4:**
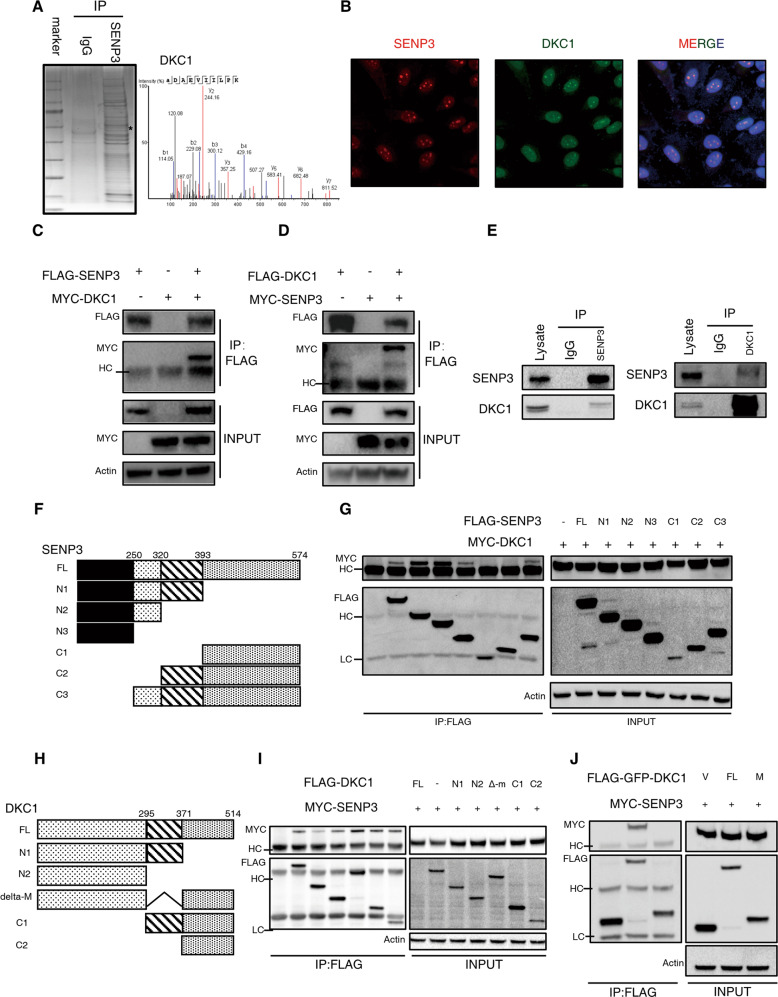
SENP3 interacts with DKC1. **A** Patu-8988t cell lysates was subjected to immunoprecipitation with control IgG or anti-SENP3 antibody, followed by commassie blue staining (left panel). The best unique peptide-spectrum matches (PSM) of DKC1 (right panel). **B** Colocalization of SENP3 and DKC1. Hela cells were transfected with expression vectors encoding Flag-SENP3 and HA-DKC1. The relative overlap of SENP3 protein and DKC1 protein was observed by fluorescence microscopy (blue: DAPI, red: SENP3, green: DKC1). Co-immunoprecipitation (co-IP) of SENP3 with DKC1. HEK293T cells expressing the indicated constructs encoding Flag-SENP3 and Myc-DKC1 (**C**) or tag-exchanged panel (**D**) were Lysed and incubated with anti-Flag gels overnight, Myc-tagged molecules co-IPed in this manner were resolved by SDS/PAGE and detected by immunoblotting with anti-Myc antibodies. The expression of SENP3 and DKC1 by transfectants (INPUT) in these studies was also confirmed by immunoblot analysis. **E** Endogenous, reciprocal co-IP of SENP3 and DKC1 in Patu-8988t cell lysate. Antibodies against SENP3 (left) and DKC1 (right) were used to pull down target proteins and their interaction partners, which was resolved by SDS/PAGE and visualized by immunoblotting. **F** Schema for generating deletion mutant constructs encoding Flag-labeled SENP3 truncates lacking specific regions. **G** Deletion mutants and a full-length SENP3 encoding construct as well as an empty vector control was co-expressed with Myc-tagged DKC1 molecules in HEK293T cells. Pull-down with anti-Flag gels and subsequent immunoblotting for Myc revealed which SENP3 variants could interact with DKC1. **H** Schema for generating deletion mutant constructs encoding Flag-labeled DKC1 truncates lacking specific regions related in Fig. 4I. **I** Each deletion mutant and a full-length DKC1 encoding construct as well as an empty vector control was co-expressed with Myc-tagged SENP3 molecules in HEK293T cells. Pull-down with anti-Flag gels and subsequent immunoblotting for Myc revealed that each DKC1 variant could interact with SENP3. **J** A Flag-GFP expression vector encoding with the middle fragment of DKC1 (amino acids 295-371, Flag-GFP-DKC1-M) was constructed and then co-expressed with Myc-tagged SENP3 molecules in HEK293T cells, as control Flag-GFP empty vector and Flag-GFP-DKC1-FL was also transfected. Pull-down with anti-Flag gels and subsequent immunoblotting for Myc revealed that the DKC1-M variant could not interact with SENP3. HC, high chains of antibodies used for IP. LC, low chains of antibodies used for IP. *in A indicates DKC1 position in stained gel.

Given that the integrated anti-tumor potency of SENP3 is enzymatically dependent, it is essential to explore its partners and potential substrates. To this end, we captured SENP3 and its associated proteins from PDAC cell lysates using the protein A/G agarose beads combined with anti-SENP3 antibodies. Subsequent mass spectrometry (MS) results identified DKC1 as a potential candidate (Fig. 4A). To verify the decisive role of DKC1 in the anti-metastasis effect of SENP3, DKC1 was knocked down (sh-DKC1) in PDAC cells, and our would healing results showed a reduced migration in these sh-DKC1 cells compared with the control (Fig. S2B). Interestingly, when wild-type SENP3 was overexpressed (S3-oe) in these sh-DKC1 cells, there were no significant differences in metastasis between these two sh-DKC1 cells with SENP3-oe or not (Fig. S2B), which implicated that SENP3 executed its regulatory effects on PDAC in a DKC1 dependent manner.

To validate our MS results, Hela cells were transfected with both Flag-tagged SENP3 and HA-tagged DKC1 constructs, the fluorescence images illustrated their colocalization in the nucleus (Fig. 4B). Additionally, SENP3 and DKC1 were co-immunoprecipitated (co-IP) when expressed ectopically in HEK293T cells (Fig. 4C, D). Furthermore, this interaction was also confirmed in primary PDAC cell lysates using a reciprocal endogenous co-IP approach, by which we found that SENP3 was able to pull down associated DKC1 proteins and vice versa (Fig. 4E). To map the domains that mediate the interaction between SENP3 and DKC1, a series of deleting mutants were expressed in HEK293T cells (Fig. 4F, H). Our co-IP assays (Fig. 4G, I, J) and simulated molecular docking results (Fig. S4) showed that two N-terminal regions (amino acids 1-250, 250-320) of SENP3 bound to DKC1, whereas both N-terminal (amino acids 1-195) and C-terminal (amino acids 371-514) regions of DKC1 were responsible for the interaction with SENP3.

### SENP3 is the deSUMOylating enzyme for DKC1, which is modified by SUMO3

**Fig. 5:**
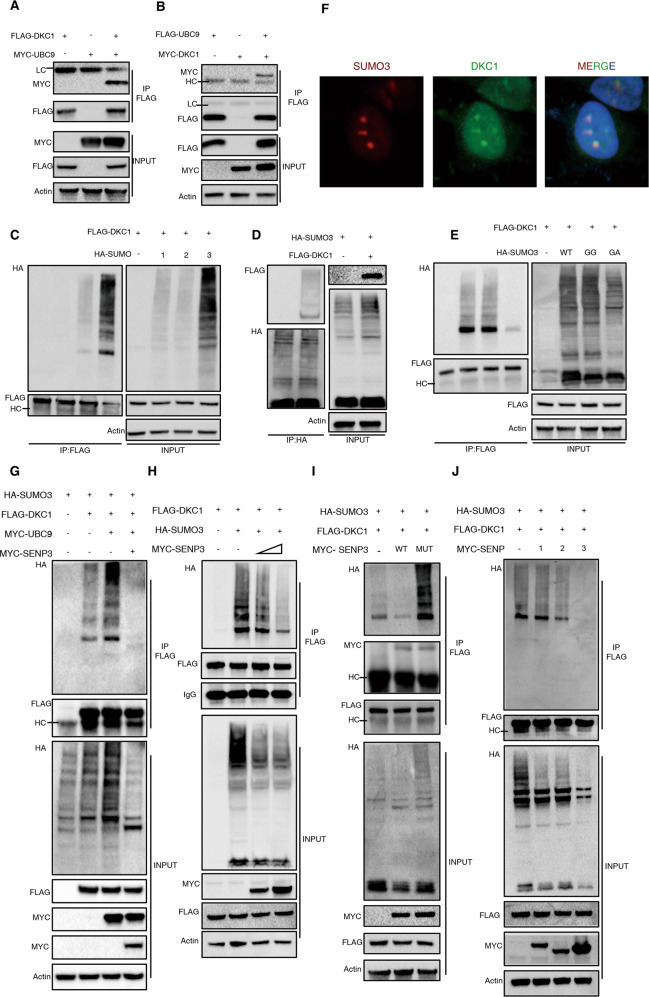
DKC1 can be SUMOylated mainly by SUMO3, which is reversed by SENP3. **A**, **B** Interaction between DKC1 and UBC9. HEK293T cells were transduced with expression vector encoding indicated molecules before lysis and co-IP test. DKC1 and UBC9 levels were determined by immunoblot assay after SDS-PAGE resolution. **C** DKC1 is majorly modified by SUMO2/3. HA-labelled SUMO1/2/3 was co-transfected into HEK293T cells with Flag-DKC1 construct. SUMOylation of DKC1 by different modifiers was detected by immunoblot after pulling down and separating with SDS/PAGE. **D** Conjugating of SUMO3 to DKC1 was confirmed by IP with anti-HA gels. **E** HEK293T cells were transfected with indicated combination of expression constructs encoding Flag-DKC1 and HA-tagged SUMO3 molecules—either wild-type(WT) or an active form(GG) or a variant with a G-to-A mutation(GA) responsible for preventing conjunction of SUMO3 to target protein. Modification of DKC1 by different SUMO3 variants was visualized by immunoblot following pulling down and separating with SDS/PAGE. **F** Colocalization of DKC1 and SUMO3. Hela cells were co-transfected with expression vectors encoding Flag-DKC1 and HA-SUMO3. The relative overlap of SENP3 protein and DKC1 protein was observed by fluorescence microscopy (blue: DAPI, red: SUMO3, green: DKC1). **G** SUMOylation levels of DKC1 were detected in presence of SENP3. HEK293T cells were transfected with a combination of indicated constructs before lysis and pull-down with anti-Flag gels. SUMOylation of DKC1 was determined by immunoblot assay with anti-HA antibodies after SDS-PAGE resolution in denaturing condition. **H** Increasing amount of expression vector encoding SENP3 decreased DKC1 SUMOylation in a dose-depend manner. The extent of DKC1 SUMOylation was detected in HEK293T cells transfected with different amounts of SENP3. **I** Assessment of SUMOylation of DKC1 upon expression of wild-type and catalytically inactive SENP3 mutants. Given combinations of constructs encoding wild-type SENP3, the functionally deficient C532S SENP3 (MUT), Flag-DKC1, and HA-labeled SUMO3 molecules were transfected into HEK293T cell for 24 h. Cells were lysed and DKC1 were recovered by pulling down using anti-Flag gels and then detected with immunoblotting with indicated antibodies. **J** Determination of SUMOylation degree of DKC1 in presence of SENP1, SENP2 or SENP3. HEK293T cells were transfected with the indicated panel, cell lysates were used to detect the SUMOylation level of DKC1 as processed in **E**. HC, high chains of antibodies used for IP. LC, low chains of antibodies used for IP.

Next, we set out to study whether DKC1 could be SUMOylated. Indeed, our co-IP assay revealed that DKC1 could interact with UBC9, the only known E2 conjugating enzyme in the SUMO cycle (Fig. 5A, B). Given the probability that the biochemical function of SUMO modification might vary according to conjugation with distinct modifiers, HA-labelled SUMO1, SUMO2, or SUMO3 was expressed ectopically with Flag-DKC1 in HEK293T cells. In Flag-DKC1 proteins precipitated with anti-Flag gels, the SUMOylation bands were detected in the presence of SUMO2 or SUMO3 (Fig. 5C), which share 97% sequence identity [29]. Additionally, SUMO3 modification of DKC1 was further confirmed by apparent shift bands detected in the purified proteins pulled down with anti-HA gels from lysates of cells expressing HA-SUMO3 and Flag-DKC1 (Fig. 5D). This shift band was readily detectable only when wild-type SUMO3 or its active form (SUMO3-GG) was expressed, but not when the SUMOylation-dead mutant (SUMO-GA [30]) was expressed (Fig. 5E). Furthermore, colocalization of DKC1 and SUMO3 in the nucleus was also illustrated by confocal microscopy (Fig. 5F).

We also sought to assess whether SENP3 could reverse the SUMOylation of DKC1. As shown in Fig. 5G, the expression of UBC9 in HEK293T cells greatly enhanced DKC1 SUMOylation, which was remarkably eliminated in the presence of SENP3. Moreover, ectopic expression of increasing amount of SENP3 constructs resulted in a dose-dependent decrease in DKC1 SUMOylation (Fig. 5H). As predicted, the C532S mutant of SENP3, which no longer has the catalytic activity, failed to decrease DKC1 SUMOylation (Fig. 5I), although it could also interact with DKC1. The specific role of SENP3 in DKC1 deSUMOylation was also verified as other deSUMOylation enzymes, SENP1 and SENP2, failed to lower the extent of DKC1 SUMOylation (Fig. 5J). we also tested the modulatory role of SENP3 in deSUMOylation of the other subunits of snoRNP proteins, the results showed that only DKC1 was regulated by SENP3 among this complex (Fig. S5). In summary, DKC1 is subjected to the modification of SUMO3, which is reversed by SENP3.

### K413, K448 and K467 are the major SUMO acceptors in DKC1

**Fig. 6:**
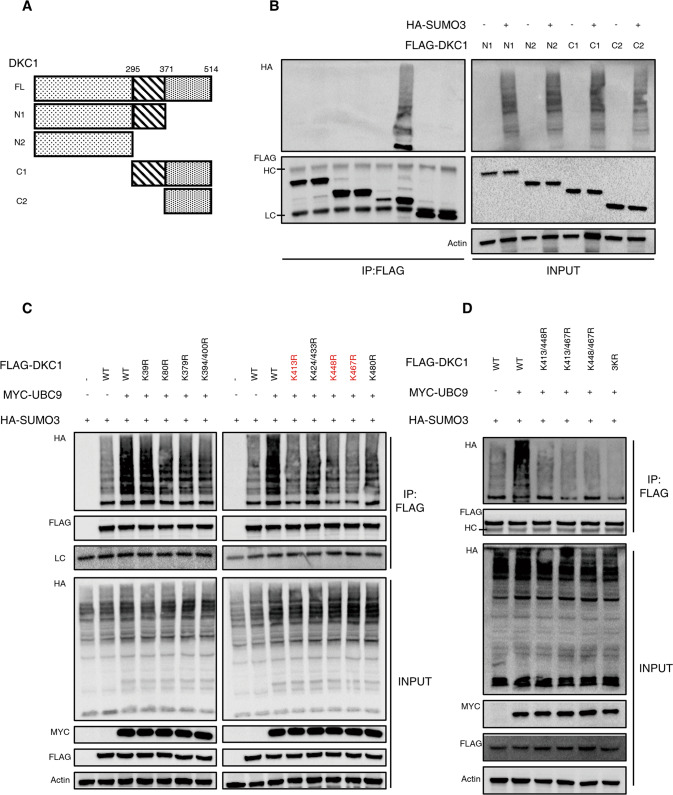
DKC1 is SUMOylated at K413, K448, K467. **A** Illustration for deletion mutant vectors encoding Flag-tagged DKC1 truncates lacking specific sequences. **B** Determination of SUMOylation among DKC1 deletion mutants. Following lysis of cells carrying the indicated deletion mutant encoding constructs with HA-SUMO3 or not, levels of SUMOylated DKC1 species in each were revealed by pulling down with anti-Flag gels and immunoblot probing for HA among the immunoprecipitated proteins. The expression of DKC1 and SUMO3 by transfectants (INPUT) in these studies was also confirmed by immunoblot analysis. **C** Immunoblot analysis of UBC9-mediated SUMOylation of wild-type and mutant DKC1 molecules. HEK293T cell lines were transfected with a normal Flag-tagged DKC1 construct, or one of constructs encoding a predicted SUMOylation-resistant mutant in which one lysine residue was replaced by arginine (K-R). These cell lines also received expression vectors encoding Myc-UBC9 and HA-SUMO molecules. Negative controls did not receive UBC9 or expressed SUMO alone. Labeled DKC1 proteins were pulled down from cell lysates with anti-Flag gels, and SUMOylated species were visualized for HA by immunoblotting. **D** HEK293T cell lines were transfected with Flag-labelled wild-type or mutant DKC1 (K413/448 R, K413/467 R, K448/467 R, all three sites mutated by K-R). Encoding vectors of Myc-UBC9 and HA-SUMO3 were also transfected into these cell lines. Negative control did not receive UBC9. Tagged DKC1 proteins were purified with anti-Flag gels, and the SUMOylation degree was illustrated by immunoblot assay with anti-HA antibodies after resolution by SDS-PAGE. HC, high chains of antibodies used for IP. LC, low chains of antibodies used for IP.

We then attempted to identify the potential target sites in DKC1 that are subjected to SUMOylation. Multiple studies have reported that SUMOylation generally takes place at the lysine residues of substrates, and the DKC1 protein contains 62 lysine residues. We therefore split the full-length DKC1 into 4 overlapping fragments, and assessed the SUMOylation status as well as subcellular distribution of each fragment (Figs. 6A, S6). Interestingly, we found only C1 (amino acids from 295 to 514) of DKC1 could be SUMOylated as indicated by the obvious smearing band (Fig. 6B, lane 6 in IP), although the fragment N1 and C2 shares part of sequences in C1. We speculated that the SUMOylated segments might contain both the E2-binding domain and the potential modified residues, which was only met by C1.

Lysine residues undergoing SUMOylation usually occur at a consensus motif, ΨKXE (Ψ represents a hydrophobic residue and X is any residue) [9]. Analysis of DKC1 with online SUMOylation predictors, GPS-SUMO and SUMOplot^TM^, identified 17 putative residues in the full length DKC1 and 9 in the C1 fragment. We then separately mutated all 9 putative lysine residues in fragment C1 along with another 2 lysine residues predicted with high scores (K39, K80) to arginine, which is resistant to SUMOylation. As before, SUMOylation was prevalent in the wild-type DKC1 pool in the presence of UBC9. However, only K413R, K448R, K467R DKC1 exhibited a mild reduction in the lower migrating band, raising the possibility that DKC1 might harbor multiple sites subjected to the modification (Fig. 6C). To test this, a set of combined mutants was constructed and then subjected to pulling down and immunoblot to assess the extent of SUMOylation. As predicted, we found that DKC1 mutated with three residues showed a relative dearth of SUMOylation as compared with its counterparts (Fig. 6D). Collectively, we identified K413, K448 and K467 as the major SUMO receptors in DKC1.

### SUMOylation promotes the stability of DKC1 and its interaction with NHP2

**Fig. 7:**
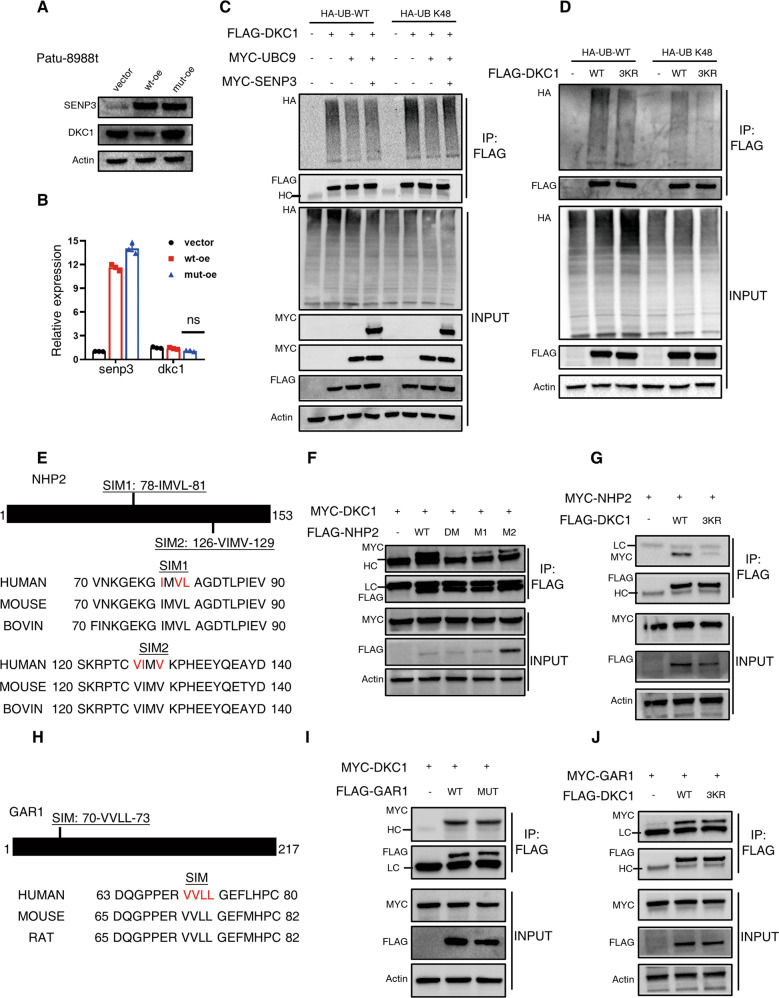
SUMOylation stabilizes DKC1 and promotes its interaction with snoRNP partners. **A** DKC1 protein levels were detected by immunoblot assay. Cell lysates from Patu-8988t cell lines infected with lentivirus expressing vector, wild-type SENP3 or catalytically deficient SENP3 were subjected to SDS-PAGE and then visualized by immunoblot using indicated antibodies. **B** DKC1 transcriptional levels were assessed by RT-qPCR. **C** Assessment of ubiquitination among DKC1 under distinct SUMOylation degrees. HEK293T cells were transfected with indicated panel, in which SUMOylation degrees was modulated by UBC9 (up) and SENP3 (down), UB WT constructs were used to reveal total ubiquitination levels in DKC1 while an UB K48 variant was responsible for detecting K48-polyubiquitination status of this protein. Negative control did not receive DKC1-encoding vectors. Cell lysates were collected after four-hour treatment with MG132 and then subjected to pulling down with anti-Flag gels. Ubiquitination levels were visualized by immunoblot (anti-HA) following SDS-PAGE in denaturing condition. **D** Determination of ubiquitination status of wild-type and mutant DKC1 molecules. HEK293T cells received empty vectors or expressing constructs encoding either wild-type or SUMOylation-resistant DKC1. These cells were transfected with UB molecules as shown in (**C**) to reveal different ubiquitination types. Cell lysates were recovered after treatment with MG132 and then subjected to IP with anti-Flag gels. Proteins were resolved by SDS-PAGE and subsequently detected by immunoblot with anti-HA antibodies. **E** Sequence alignments of two conserved SUMO-interacting (SIM) sites in NHP2 among different species. The indicated amino acids in red (I/L/V) within the SIM were mutated to alanine in NHP2 mutants. **F** Interaction between DKC1 and NHP2 variants was assessed. HEK293T cells were transduced with expression vectors encoding wild-type NHP2 or mutated NHP2 in one or two SIM sites. These cells were co-transfected with MYC-labelled DKC1 construct and then subjected to co-IP test. After resolution in SDS-PAGE, DKC1 and NHP2 levels were revealed by immunoblot assay with anti-MYC and anti-Flag antibodies respectively. **G** Interaction between NHP2 and DKC1 variants was tested. Wild-type and SUMOylation-resistant (3KR) DKC1 were transfected into HEK293T cells, which were epigenous expressing MYC-tagged NHP2 by plasmid transduction. An empty vector was used as control. The co-IP test was carried out using lysates of these transfected cells, and the protein levels were detected by immunoblot with indicated antibodies. **H** Sequence alignment of SIM site in GAR1 among different species. The highlighted red amino acids were mutated to alanine. **I** Mutation of SIM in GAR1 did not disrupt the interaction between DKC1 and GAR1. Wild-type or mutated GAR1 was co-transfected into HEK293T cells with Myc-tagged DKC1. DKC1 was detected in the pooled complexes IPed by anti-Flag gels by SDS/PAGE resolution and immunoblot. **J** GAR1 could comparably interact with SUMOylation-resistant (3KR) DKC1. Interaction between GAR1 with wild-type or SUMOylation-resistant DKC1 was assessed by co-IP assay. HEK293T cells were transfected with Myc-tagged GAR1 and Flag-tagged DKC1 variants. After being pulled down with anti-Flag gels, the associated GAR1 was visualized by SDS/PAGE and immunoblot assay. HC, high chains of antibodies used for IP. LC, low chains of antibodies used for IP. Unpaired *t* test, ns, no significance.

Similar to other types of post-translational modification, the consequences of SUMOylation are substrate specific. Initially, we sought to investigate whether DKC1 SUMOylation may alter its protein stability. The protein abundance of DKC1 was decreased in PDAC cells expressing wild-type SENP3, but remained unchanged in cells expressing catalytically inactive DKC1 (Fig. 7A). Moreover, there were no significant differences at the transcriptional level among those cells (Fig. 7B), which implicated that SENP3-mediated deSUMOylation rendered to protein instability of DKC1. To shed light on the underlying mechanism, the extent of DKC1 ubiquitination, especially K48-type polyubiquitination, was assessed under different SUMOylation status. The results showed that cells transfected with UBC9 exhibited a reduced DKC1 K48-ubiquitination level. This effect, however, was diminished when SENP3 was co-transfected (Fig. 7C). Interestingly, we also found the K48-ubiquitination extent of DKC1 mutant (mutated at SUMOylation sites by K-to-R) was evidently decreased compared to the wild-type DKC1 (Fig. 7D), which indicated that ubiquitination competed with SUMOylation at the same lysine residue(s) in DKC1.

SUMOylated proteins can bind noncovalently to other proteins via SUMO interaction motifs (SIMs) [31], which are composed of 3-4 consecutive hydrophobic amino acids (ΨΨΨΨ or ΨxΨΨ) [32]. The online bioinformatics platform (GPS-SUMO) was used to predict the putative SIMs in other components of box H/ACA snoRNP group and we found two SIMs in NHP2 (Fig. 7E) and one in GRA1 (Fig. 7H). Mutation of both two SIMs (substitution of hydrophobic amino acids with alanine) in NHP2 strongly abrogated the interaction between DKC1 and NHP2, although single mutation slightly disrupted their association (Fig. 7F). This notion was further confirmed by reduced NHP2 protein pulled down together with SUMOylation-deficient DKC1 in comparison with its counterpart (Fig. 7G). Furthermore, we also compared the interaction between DKC1 and NHP2 in PDAC cells with over-expression of SENP3 or not, and the results showed that SENP3-mediated deSUMOylation of DKC1 indeed disrupted the association of these two molecules (Fig. S7). However, this SIM-mediated interaction was not recognized between DKC1 and GAR1 (Fig. 7I, J).

### DKC1 reversed the anti-metastasis effect of SENP3 and is correlated with poor outcomes among PDAC patients

**Fig. 8:**
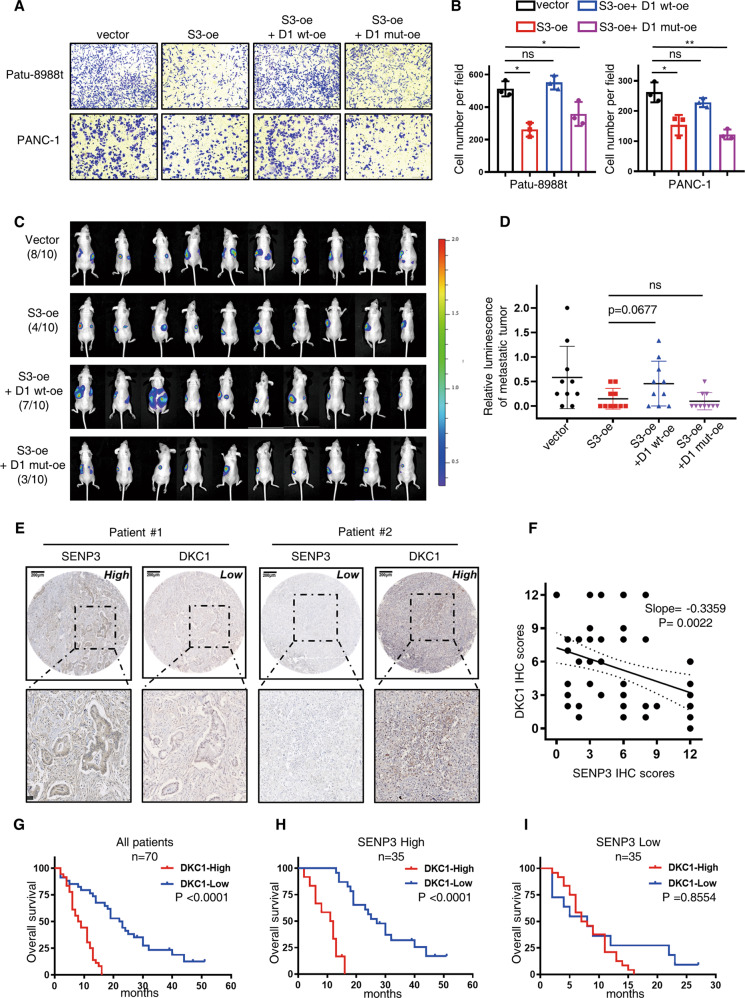
Wild-type DKC1 reverses SENP3-mediated anti-metastasis effects and is associated with poor prognosis in PDAC patients. **A**–**D** The effects of wild-type DKC1 and SUMOylation-resistant DKC1 (3KR) overexpression on PDAC cell lines with overexpressing SENP3 were determined by transwell assay as well as in vivo migration imaging. Wild-type DKC1 overexpression suppressed the anti-metastasis effects of SENP3, but mutated DKC1 with 3 K-R mutant sites could not. unpaired *T* test. **E**, **F** DKC1 expression was detected in the same PDAC tissue microarray panel shown in Fig. 1 and the correlation between SENP3 and DKC1 at protein level was analyzed. Representative images were shown in **E**, linear-regression analysis based on IHC scores was presented in **F**. **G**–**I** Overall survival based on DKC1 expression among all patients or in stratified groups according to SENP3 expression. log-rank test. ns, no significance, **p < 0.01, ***p < 0.001, ****p < 0.0001.

To confirm that the function of SENP3 was exerted through reducing the expression of DKC1 and abrogating the interaction between snoRNP proteins, we overexpressed wild-type DKC1 (S3-oe+ D1 wt-oe) or SUMOylation-resistant DKC1 (S3-oe+ D1 mut-oe) in the cells with increased expression of SENP3 (S3-oe) (Fig. S8A). As expected, wild-type DKC1 reversed the inhibitory function of SENP3 on cellular metastasis in vitro (Figs. 8A, B, S8B). SUMOylation-deficient DKC1, however, showed little effect on the inhibition of tumor migration upon SENP3 overexpression (Figs. 8A, B, S8B). Consistently, similar results were observed in the in vivo metastasis models as indicated by the improved metastatic rate only in DKC1 wt-oe group, and the relative intensity of liver foci also slightly increased in this group (*p*-value = 0.0677). (Fig. 8C, D).

To better characterize the role of DKC1 in PDAC, we determined the protein expression of DKC1 in the tissue microarray mentioned above. Consistent with previous findings, DKC1 was negatively correlated with SENP3 at the protein level (Figs. 8E, F, S8C), although no correlation was detected in the transcriptional level analyzed with three GSE databases (Fig. S8D-F). Next, we found the association of the high expression of DKC1 with a poor prognosis in PDAC patients (Fig. 8G). Interestingly, when patients were stratified by the expression of SENP3, DKC1 lost its role as an indicator for poor outcomes among patients with lower expression of SENP3 (Fig. 8H, I), implicating that SUMOylated DKC1 might exert a more robust pro-tumor effect.

## Discussion

The conjugation of small ubiquitin-like modifiers (SUMOs) to target proteins, entitled as SUMOylation, takes place through an enzymatic cascade composed of a dimeric SUMO-activating enzyme (E1, SAE1/SAE2), a single modifier-conjugating enzyme (E2, UBC9), and a modest number of E3 ligases. In humans, SUMO2 and SUMO3 share 97% sequence similarity, while SUMO1 and SUMO2/3 have only 53% similarity in sequence [10–12]. A set of SUMO-specific proteases (SENPs) facilitate the reversibility of SUMO pathway by deconjugating SUMOs from substrate proteins [33]. In addition, SENPs are responsible for priming of precursor SUMO proteins by cleaving their carboxyl termini to expose the Gly-Gly motif ready for conjugation [34]. Functionally, SUMOylation has been reported to be involved in various cellular processes such as cell cycle progression and DNA damage response. As a result, SUMO pathway is considered to play critical roles in different types of cancer [13, 14]. Indeed, multiple amounts of literature have demonstrated that the SUMO system was involved in tumor progression and associated with the prognosis of patients suffering from various tumor types, including PDAC [15, 16, 27]. The specific role of the SUMO system in PDAC metastasis, however, was little evaluated. In our study, an in vivo metastasis model was used to screen 7 SENPs and we found that SENP3 knockdown in two PDAC cell lines promoted liver metastasis of PDAC (Fig. 1A, S1A–C). Interestingly, further study unveiled the unique effect of SENP3 in modulating tumor invasion but not in cell proliferation (Figs. 2, 3, S2A), which was largely reported to be regulated by the SUMO pathway. We attributed this to the molecule-specific nature in this type of tumor. Collected, our research may broaden the application area of modulating the SUMO pathway to combat PDAC.

Although others have documented that DKC1 could be SUMOylated [35], the mechanism responsible for the regulation of DKC1 by SENP3 remains to be fully deciphered. Here we report that SENP3 colocalizes with DKC1 and catalyzes the deSUMOylation of DKC1, which accepts SUMO molecules at K413, K448 and K467 (Figs. 4–6). SENP3-mediated deSUMOylation of DKC1 abates the protein stability, and its interaction with NHP2, which therefore renders decreased cellular pseudouridine level (Fig. S9). Increasing evidence has suggested the oncogenic role of DKC1 in a variety of human tumors. Via genome-wide shRNA screening, Guangyuan Kan and his colleagues [26] identified DKC1 as a molecular driver in colorectal cancer progression and they then proved the therapeutic efficiency of synergistic inhibition of DKC1 and MEK1/2 in fighting tumor. Moreover, the expression of DKC1 was demonstrated to be elevated in hepatocellular carcinoma (HCC) and associated with a poor prognosis of HCC patients [25]. However, the effects of DKC1 in PDAC remain to be studied. Our data showed that the expression of DKC1 was increased in PDAC and associated with poor outcomes in patients (Fig. 8E, G). Interestingly, the prognostic value of DKC1 was not significant in patients with low expression of SENP3 (Fig. 8I), indicating that SUMOylation statue plays an important role in the DKC1-mediated pro-tumor effect. Given that stratification is considered as a promising strategy in fighting PDAC for the sake of heterogeneity [36], our findings provide basic evidence for application of DKC1 inhibition in PDAC patients with different expression of SENP3.

The crosstalk between SUMOylation and ubiquitination has been largely reported [37–39], as increasing proteins are found to serve as substrates for both SUMO and ubiquitin, even targeted at the same lysine residue [40–42]. In the NF-κB signaling pathway, ubiquitination of the NF-κB inhibitor, IκBα, triggers signaling translocation into the nucleus and hence activates its target genes. However, SUMOylation was shown to block IκBα ubiquitination by direct competition for the same lysine, K21 [40]. On the other hand, Xiao-xin Sun et al. [41] reported that SENP1 deSUMOylated and stabilized c-Myc by inhibiting ubiquitination-mediated degradation, which indicated that these two modification types synergically regulated the stability of c-Myc. In the context of DKC1, the protein level of DKC1 was reduced in PDAC cells overexpressing SENP3 (Fig. 7A), and the negative correlation between DKC1 and SENP3 was confirmed in PDAC specimens (Fig. 8E, F). To shed light on the relationship between SUMOylation and ubiquitination in DKC1 degradation, the extent of K48 polyubiquitination, well known for triggering proteasome-mediated degradation, was tested through pull-down and immunoblot. Our results showed that SENP3 could reverse UBC9-mediated reduction of DKC1 ubiquitination (Fig. 7C). Further findings implicated that SUMOylation competed with ubiquitination for the same sites in DKC1 (Fig. 7D).

In summary, we have revealed a previously uncovered role of SENP3 in PDAC. We found that the expression level of SENP3 was markedly decreased in PDAC tissues and correlated with a favorable prognosis in PDAC patients. In addition, overexpression of SENP3 in PDAC cell lines reduced the invasion ability both in vivo and in vitro, which was dependent on its catalytic activity. Mechanistically, SENP3 interacted with DKC1 and mediated deSUMOylation of this protein at three different lysine residues. DeSUMOylated DKC1 was unstable and likely to lose its interaction with NHP2, thus disrupting the function of snoRNPs. We also confirmed that DKC1 was able to reverse the anti-metastasis effect of SENP3 and was associated with poorer outcomes in patients with PDAC. Interestingly, among patients with low expression of SENP3, there were no prognostic differences between patients with different expression of DKC1. These findings thus highlighted the pivotal role of SENP3/DKC1 axis in the progression of PDAC.

## Materials and methods

### Patients

Ten PDAC patients, who were diagnosed by histology and underwent pancreatectomy at the First Affiliated Hospital of Sun Yat-Sen University (Guangzhou, China), were involved in this study, which was approved by the Ethical Committee of the First Affiliated Hospital of Sun Yat-Sen University. The sample proteins were harvested using the minute^TM^ total protein extraction Kit (Invent, USA) as previously described [43], and subsequently subjected to western blot assay.

### Animal experiments

BALB/c-nude mice (aged 4-6 weeks) were purchased from the GemPharmatech Co., Ltd (Nanjing, China) and maintained under specific pathogen-free condition. Animal protocols were approved by the Animal Care and Use Committee of Sun Yat-Sen University. Previously described protocol [43] was used to perform the orthotopic xenograft. In brief, after anesthetization, the left flank of the mice was cut open, and the spleen was exteriorized to access the pancreas. Prepared cells were orthotopically injected into the head of the pancreas. Fluorescence signals and metastatic status were monitored using the IVIS imaging system (PerkinElmer, USA) for 8 weeks.

### Plasmid construction and lentivirus production

pcDNA3.1 Flag, HA, Myc vectors were used to make expression plasmids. Full-length SENP3, DKC1, UBC9, pro-SUMO1/2/3, ubiquitin, NHP2 and GAR1 were obtained from cDNA of PDAC cells by polymerase chain reaction (PCR) using specific primers and validated by sequencing. Site-directed mutagenesis was carried out through overlap PCR or Fast Mutagenesis Kit V2 (Vazyme Biotech, China) according to manufacturer’s instruction. The fusion segment of DKC1 was obtained by overlap PCR. Expression plasmids for tag-labelled genes were generated by inserting indicated genes into pcDNA 3.1 vectors and then validated by sequencing.

For establishing knockdown cell lines, pLKO.1 vector was used to express the shRNA against the indicated human genes. The shRNA sequences for each gene were listed in supplementary information. For the establishment of overexpressing cell lines, lentiviral vector pLVX infused with puromycin or GFP for election was utilized and indicated genes were inserted into pLVX vectors and confirmed by sequencing. To produce lentiviral particles, these lentiviral vectors mentioned above were co-transfected with PLP1, PLP2 and VSVG into HEK293T cells using the PEI reagent (Yeasen Biotechnology, China). 48 h after transfection, the virus particles were collected and filtered through a 0.45um filter. Cells were infected with virus in the present of polybrene (8 mg/ml, Sigma-Aldrich) and selected with puromycin.

### Western blot analysis

Cell lysates were collected in RIPA lysis buffer (50 mM Tris-HCl pH 7.4, 1 mM EDTA, 0.25% deoxycholic acid disodium salt, 1% NP40, 150 mM NaCl, 0.1% SDS) supplemented with protease inhibitor (TargetMol Chemicals, USA) and PMSF. After quantification by BCA assay, 30 μg of protein lysates were separated by SDS/PAGE and then transferred to a PVDF membrane (Bio-Rad, USA). After 1 h blockade, the membranes were incubated with appropriate antibodies overnight at 4 °C and then washed with TBST three times. Following incubation with secondary antibody conjugated with horseradish peroxidase for 1 h at room temperature, the protein-specific bands were detected using a chemiluminescence reagent (ECL, Millipore, USA).

### Immunoprecipitation (IP)

PBS-washed Cell pellets were incubated with IP lysis buffer (25 mM Tris-HCl pH 7.4, 150 mM NaCl, 1% NP-40, 1 mM EDTA, 5% glycerol) containing protease inhibitor and PMSF for 30 min at 4 °C. To purify tag-labelled protein, appropriate cell lysates were incubated with washed anti-Flag gels (Sigma-Aldrich, cat# A2220) or anti-HA gels (Sigma-Aldrich, cat# E6779), rotating overnight at 4 °C. The agarose beads were then washed three times with IP lysis buffer, and the precipitated proteins were collected at 95 °C for 5 min using 3x loading buffer. In immunoprecipitation of primary cells, cell lysates were incubated with antibodies against SENP3 or DKC1, rotating overnight at 4 °C. Washed protein A/G agarose beads (40ul, Santa Cruz, cat# sc-2003) was subsequently added to each sample and rotated for another 2 h at 4 °C. The elution procedures were similar to IP with gels. The immunoprecipitated complexes were then subjected to western blot analysis.

### SUMOylation and ubiquitination assays

To test the SUMOylation statues of target protein, HEK293 T cells were cultured in 6 cm plates and transfected with indicated plasmids. Whole-cell lysates were collected by lysing in SUMO lysis buffer (25 mM Tris-HCl pH 7.4, 150 mM NaCl, 1.0% (wt/vol) SDS, 20 μM NEM, 1 mM EDTA, 5% glycerol) containing protease inhibitor and PMSF. After boiling for 10 min at 95 °C, protein was centrifuged (13,500 rpm, 15 min, 4 °C) and then quantified with BCA assay. Equal amount of the protein concentrations was seemed as INPUT sample, whereas the remaining part was diluted with IP lysis buffer at 1:10 and then subjected to pull-down with anti-Flag or HA gels. Immunoprecipitated proteins were recovered in 3x loading buffer, and then visualized by western blot.

For ubiquitination assay, transfected HEK293T cell was incubated with MG132 (20 μM for 4 h) before lysis. UB lysis buffer (25 mM Tris-HCl pH 7.4, 150 mM NaCl, 1.0%(wt/vol) SDS, 20 μM MG132, 1 mM EDTA, 5% glycerol) containing protease inhibitor and PMSF was used to extract the whole-cell lysates, subsequent procedures were like SUMOylation assay.

### Immunofluorescence staining

Hela cells were cultured on coverslips in 6-well plates and transfected with indicated plasmids. Washed cells were fixed with 4% paraformaldehyde, permeabilized in 0.1% Triton X-100, and then washed with ice-cold PBS three times. The cells were incubated with the indicated primary antibodies for one hour followed by incubation with Alexa Fluor secondary antibodies (Thermo Fisher, USA).

### Bioinformatic analysis and online platforms

Three gene expression profiles (GSE15471, GSE16515, GSE32676) were downloaded from the Gene Expression Omnibus (GEO) database, the expression of senp3 and dkc1 in tumors was extracted and used to analyze the correlation between these two molecules at the mRNA level. The gene expression profiling interactive analysis (GEPIA2, http://gepia2.cancer-pku.cn) was used to demonstrate the role of distinct SUMO specific peptidases in predicting the prognosis of PDAC patients. The cBioPortal web (http://cbioportal.org), which is based on TCGA database, was used to illustrate the distribution of mutations of KRAS and TP53 in PDAC patients with different expression of senp3. To predict the potential SUMOylated residues in DKC1, online bioinformatic platform GPS-SUMO (http://sumosp.biocuckoo.org) and SUMOplot^TM^ (https://www.abcepta.com/sumoplot) were used. To predict the putative SUMO interaction motifs (SIMs) in snoRNP proteins, the GPS-SUMO was used.

### Statistical analysis

Values were presented as the mean ± standard deviation (SD) and statistical analysis was performed using SPSS software 24.0 (USA) or GraphPad Prism 8.0 (USA). The results shown were representative data from three independent experiments. Statistical differences between two groups were determined using unpaired, two-tailed student’s test. For comparison among multiple groups, the ANOVA test was used. The Chi-Square test was used for testing the differences between categorical variables. Fisher’s exact test was used when the number of variables was lower than 5. The survival curves were obtained by the Kaplan–Meier method and compared using the log-rank test. Linear-regression analysis was used to determine the correlation between SENP3 and DKC1 expression level in human specimens as well as in the GEO databases. In general, *P* value < 0.05 was considered significant and are indicated as follows: ^*^P < 0.05, ^**^P < 0.01, ^***^P < 0.001, ^****^P < 0.0001, ns: no significance.

## Supplementary information

check list

Supplementary Figure 1

Supplementary Figure 2

Supplementary Figure 3

Supplementary Figure 4

Supplementary Figure 5

Supplementary Figure 6

Supplementary Figure 7

Supplementary Figure 8

Supplementary Figure 9

Supplementary Figure 10

Supplementary Figure 11

supplementary information

uncropped original western blots

## Data Availability

All data responsible for evaluating the conclusions in the paper are presented in the paper and/or the Supplementary Materials. The datasets used and analyzed during the study are available from the corresponding author on reasonable request.
