# Supplementary figures and images for: SUMO specific peptidase 3 halts pancreatic ductal adenocarcinoma metastasis via deSUMOylating DKC1
Source: Cell Death Differ. 2023 May 15;30(7):1742–56. doi: 10.1038/s41418-023-01175-4 (PMC10307871; doi:10.1038/s41418-023-01175-4)

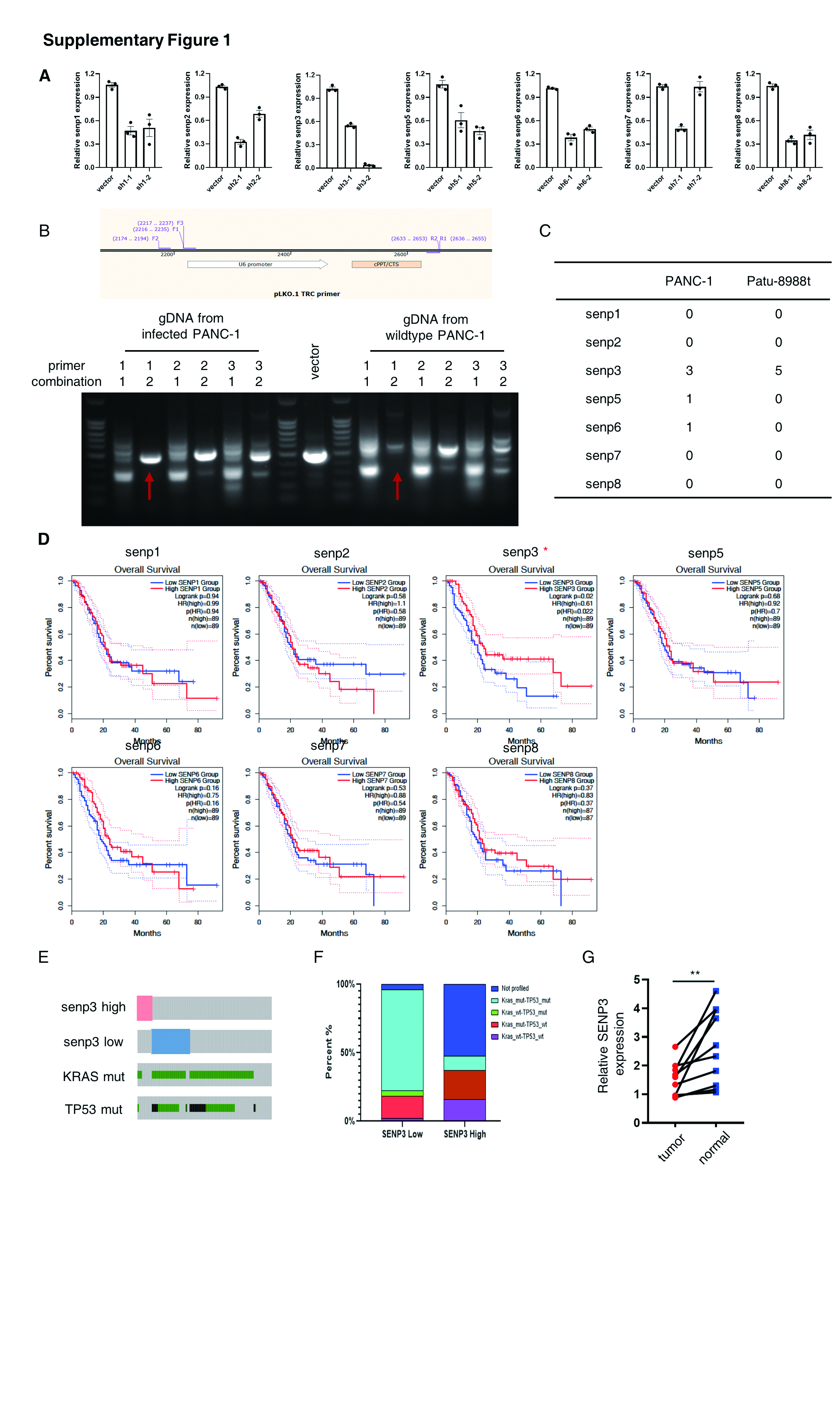

Supplement: Supplementary file 2 — Supplementary Figure 1 [file 41418_2023_1175_MOESM2_ESM.tif]

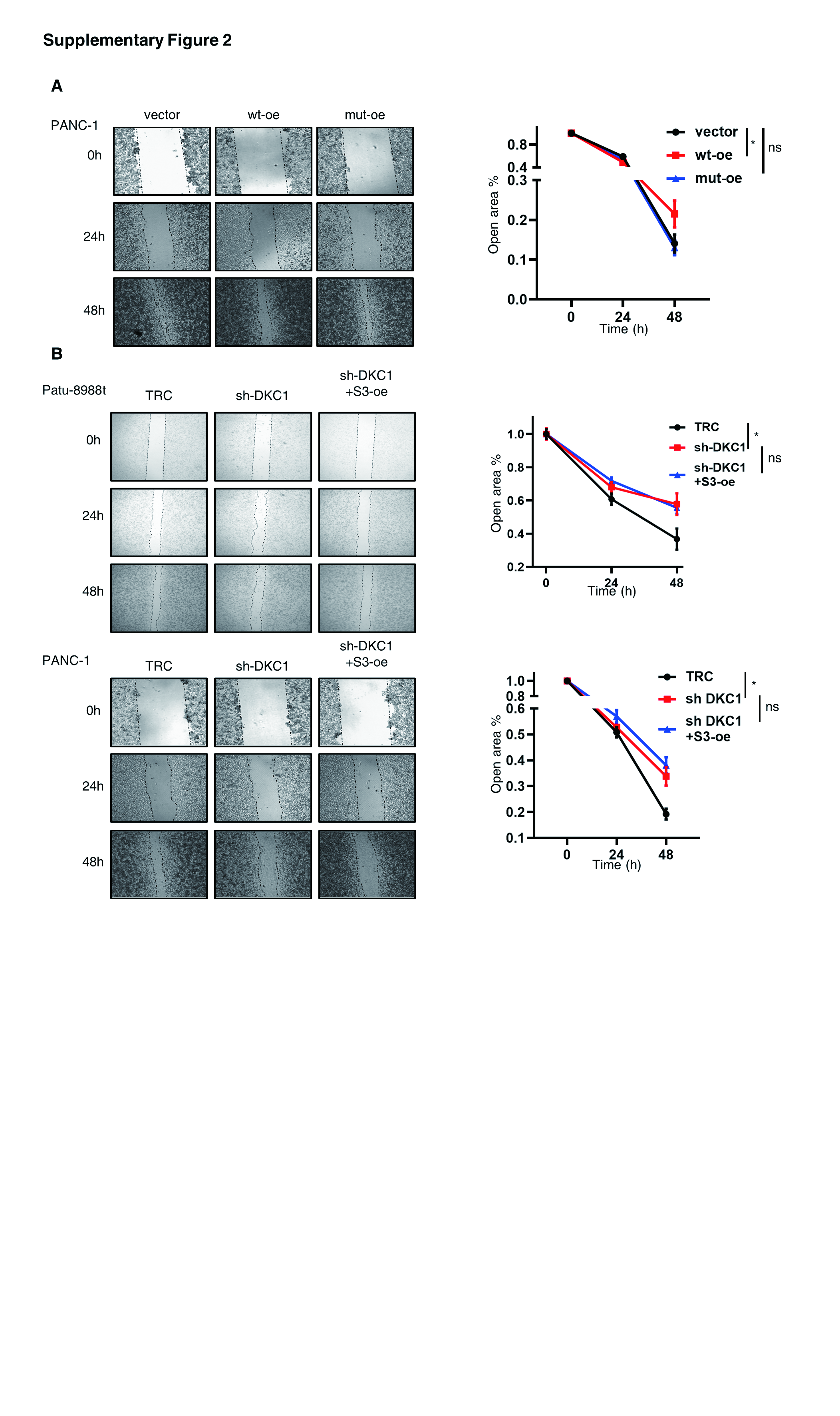

Supplement: Supplementary file 3 — Supplementary Figure 2 [file 41418_2023_1175_MOESM3_ESM.tif]

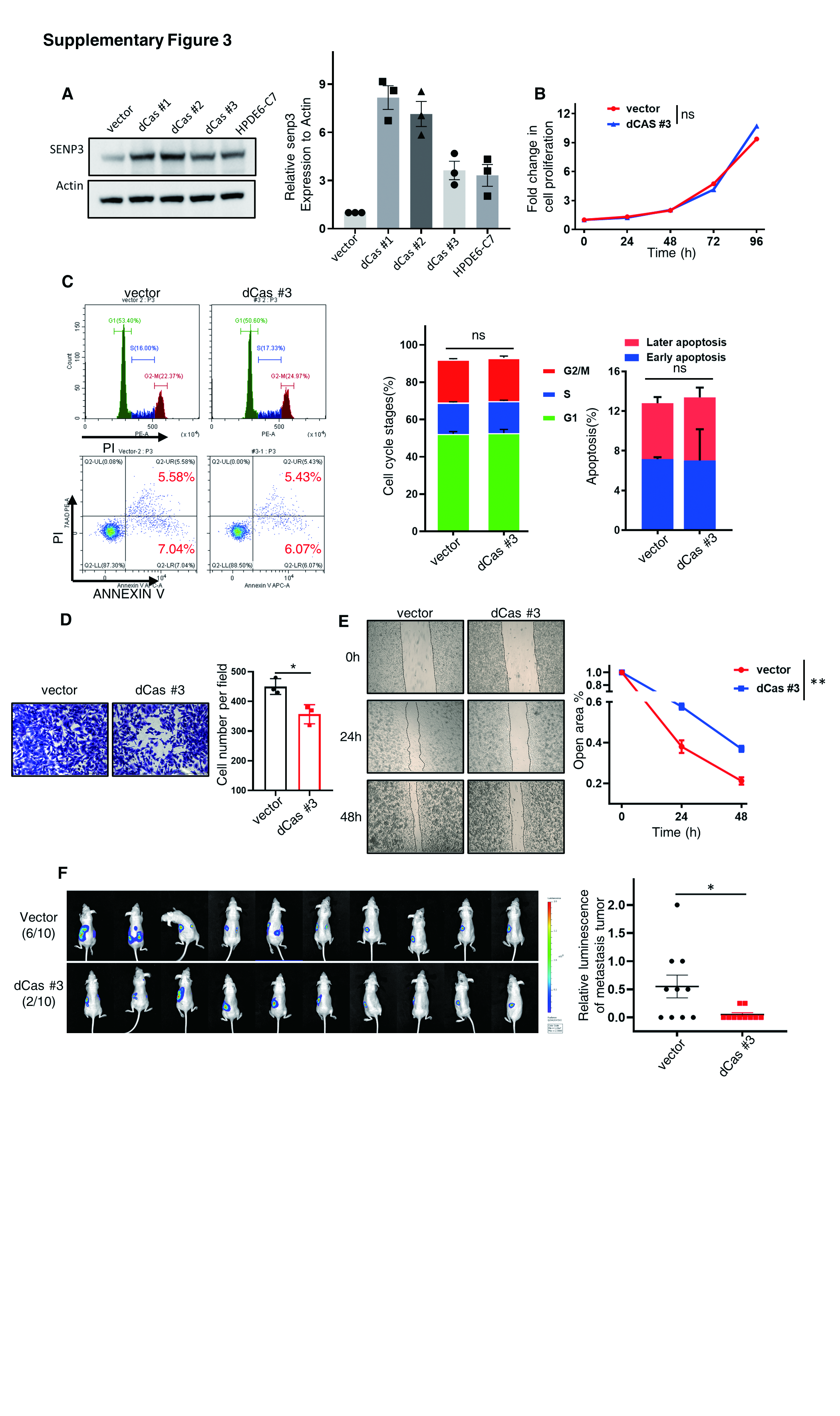

Supplement: Supplementary file 4 — Supplementary Figure 3 [file 41418_2023_1175_MOESM4_ESM.tif]

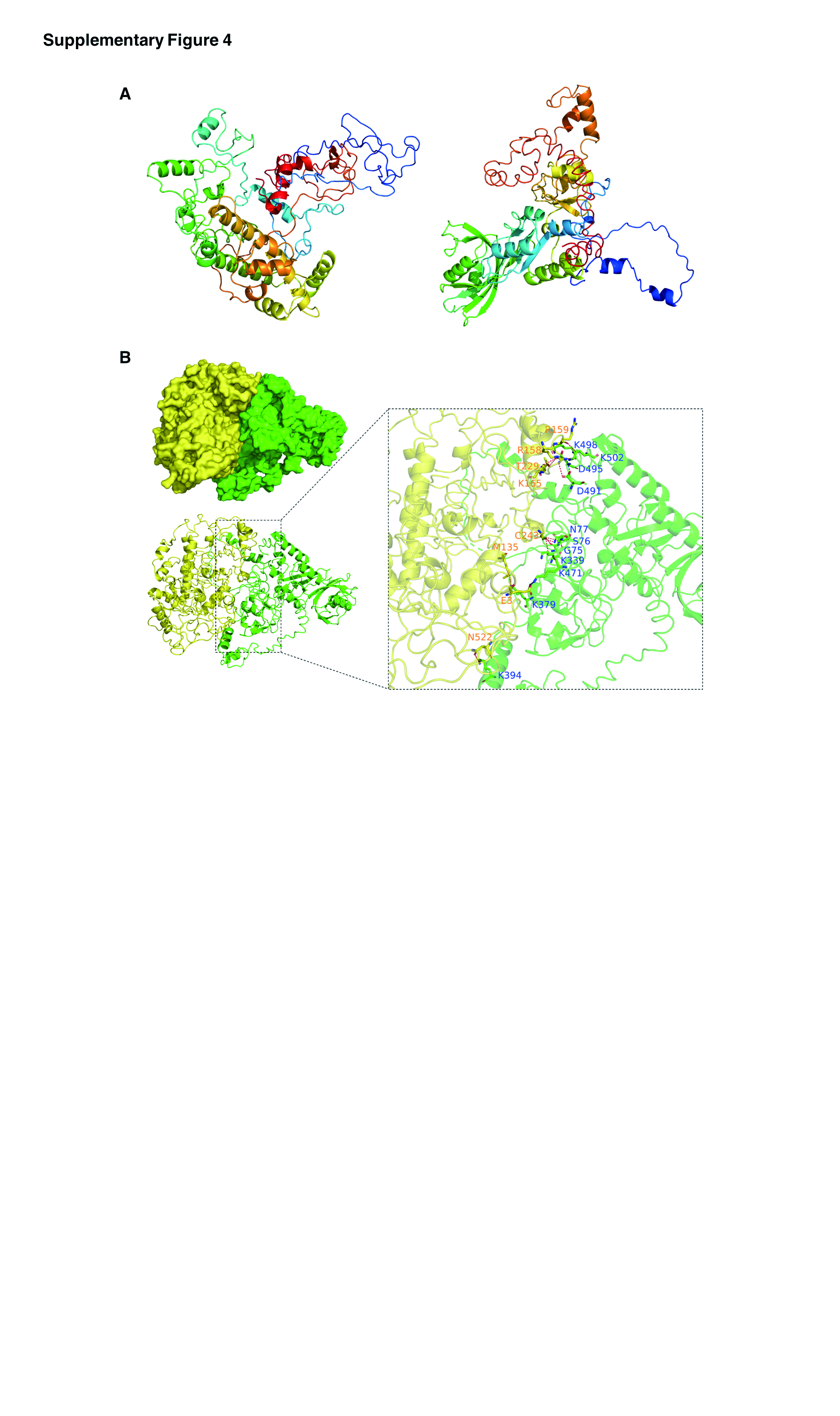

Supplement: Supplementary file 5 — Supplementary Figure 4 [file 41418_2023_1175_MOESM5_ESM.tif]

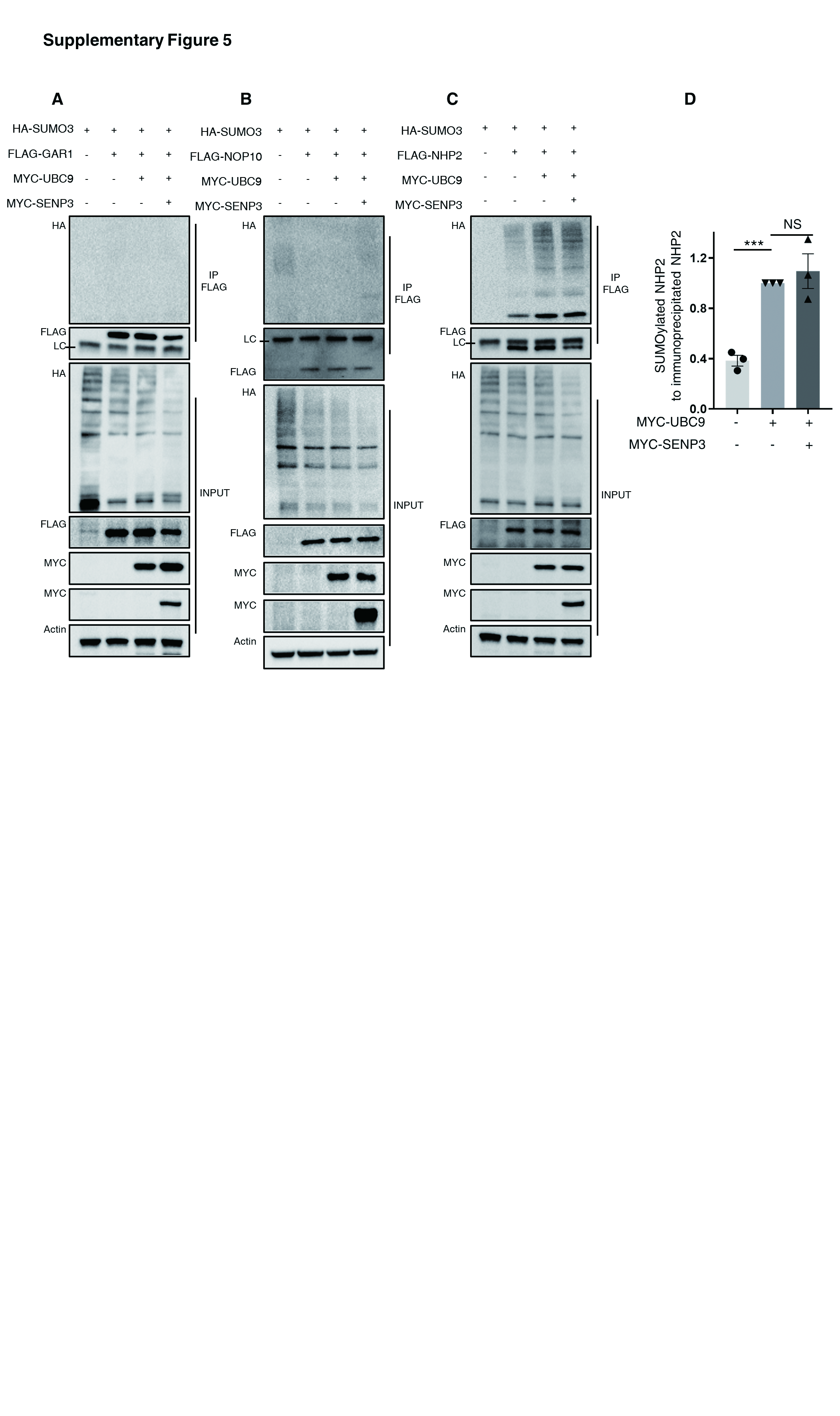

Supplement: Supplementary file 6 — Supplementary Figure 5 [file 41418_2023_1175_MOESM6_ESM.tif]

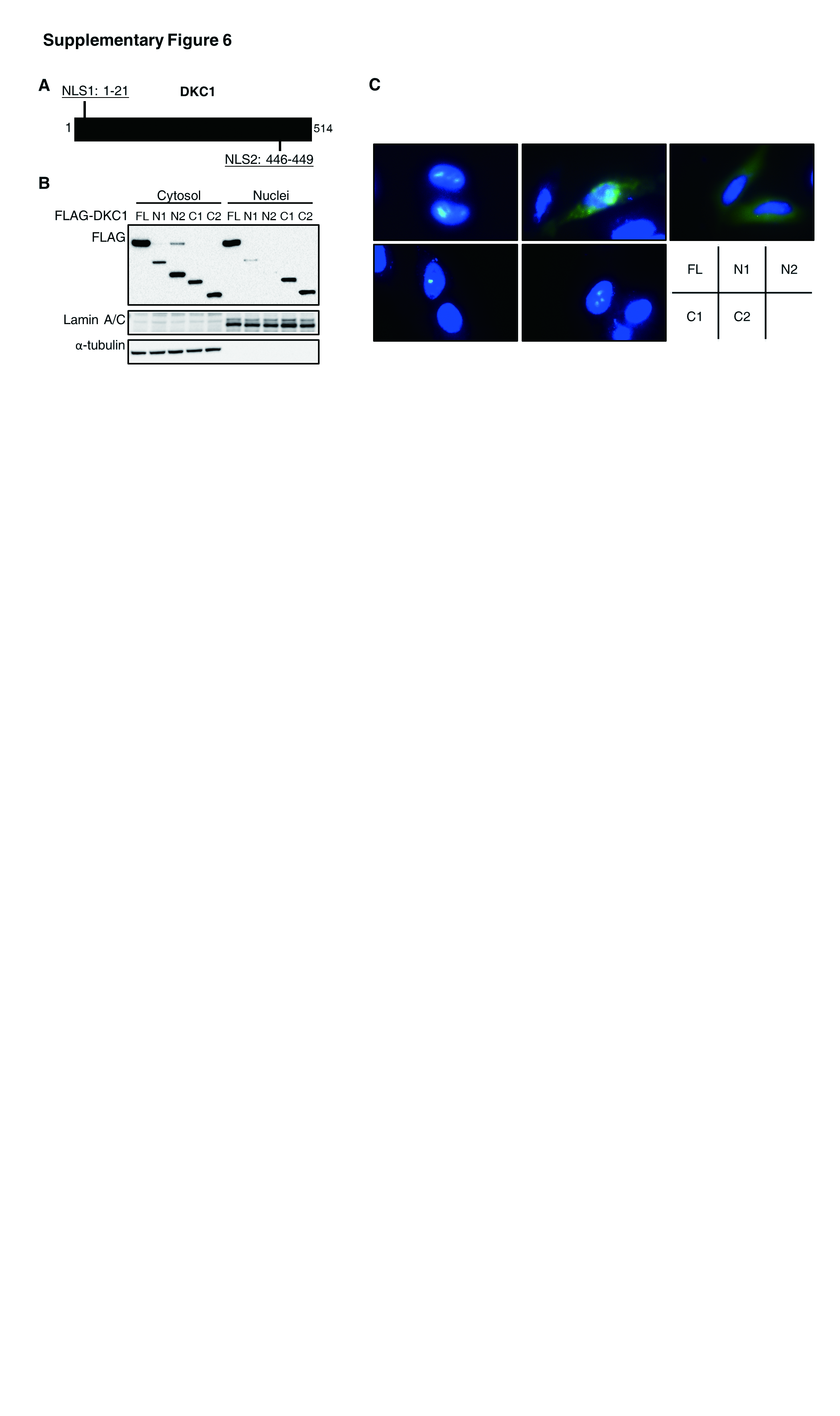

Supplement: Supplementary file 7 — Supplementary Figure 6 [file 41418_2023_1175_MOESM7_ESM.tif]

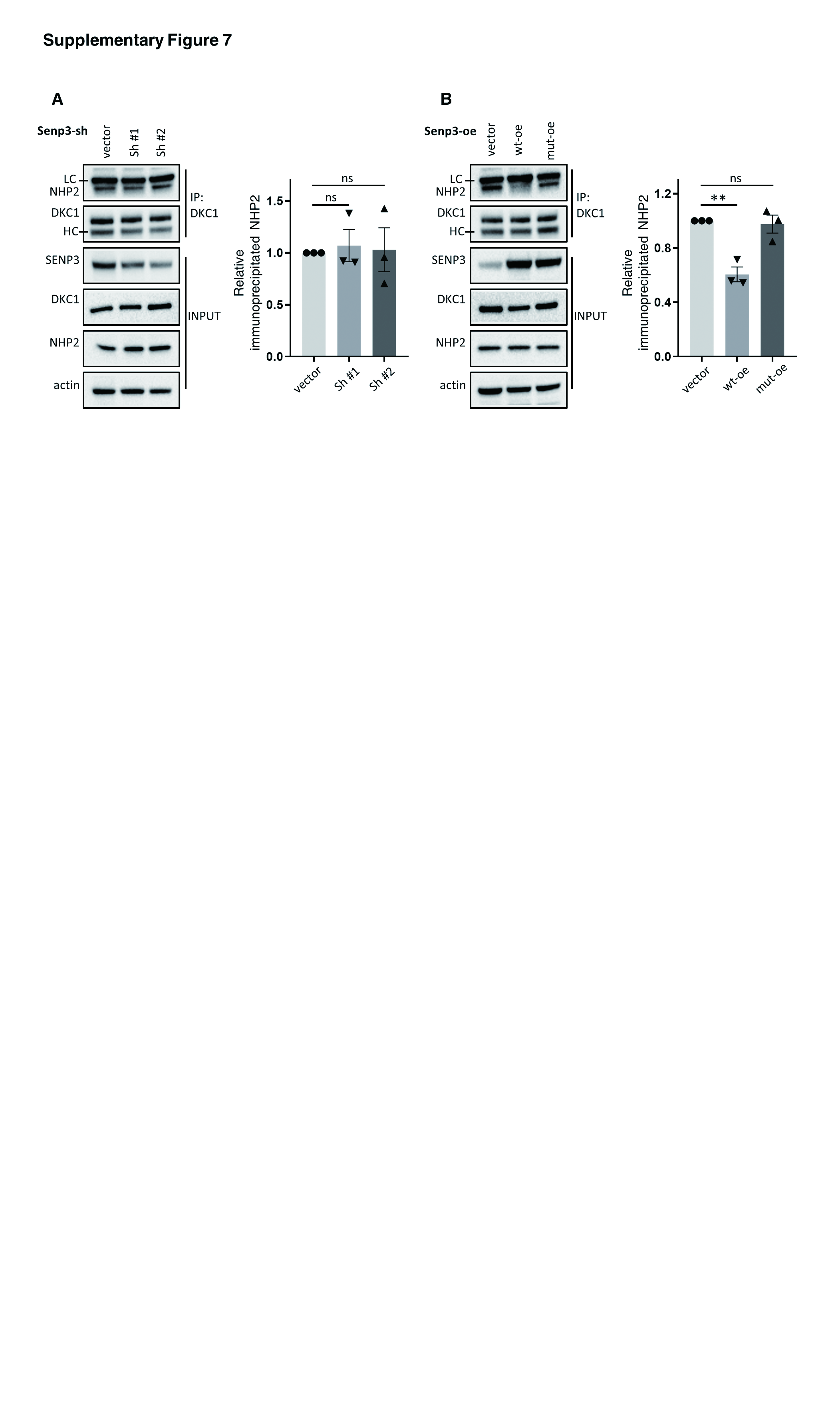

Supplement: Supplementary file 8 — Supplementary Figure 7 [file 41418_2023_1175_MOESM8_ESM.tif]

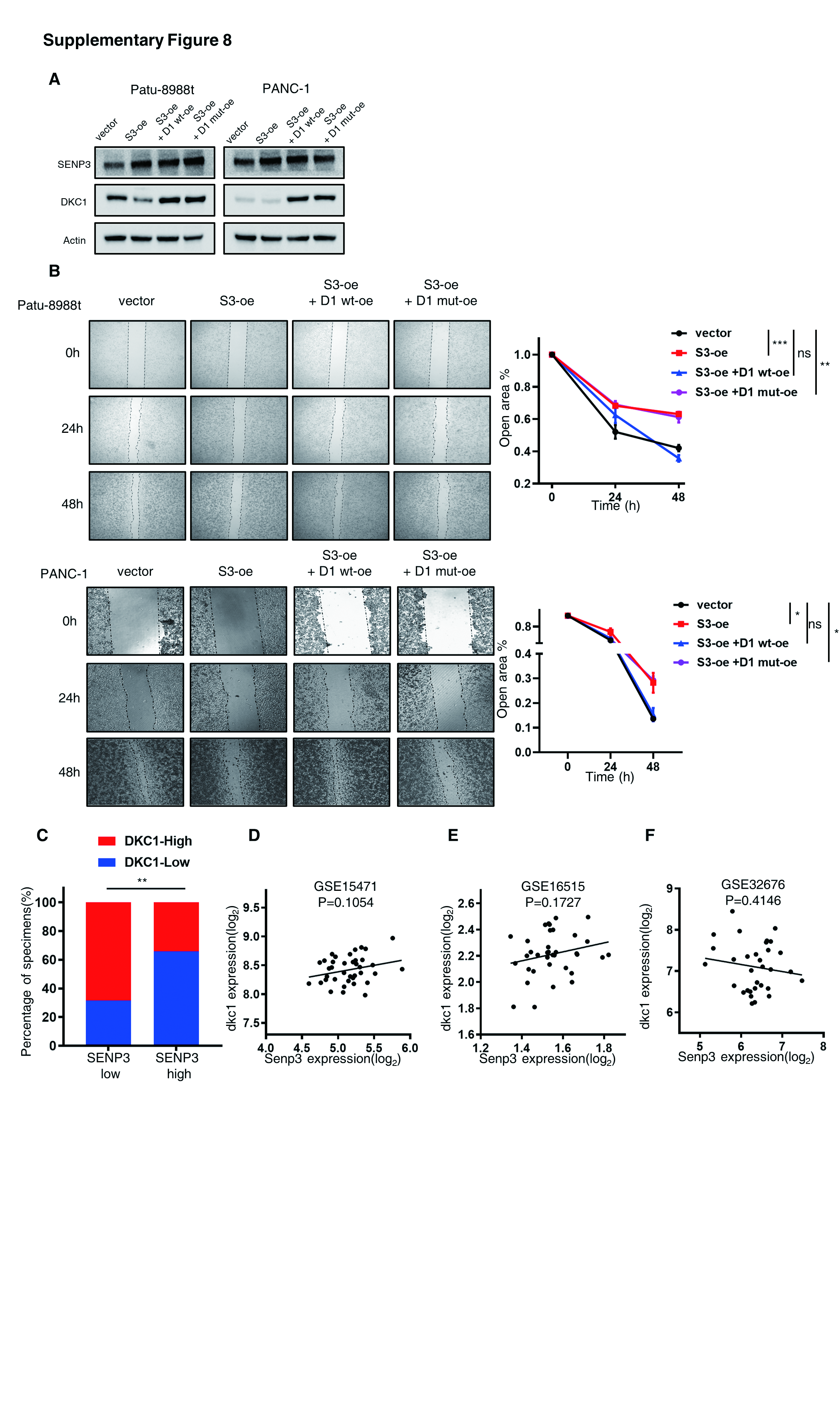

Supplement: Supplementary file 9 — Supplementary Figure 8 [file 41418_2023_1175_MOESM9_ESM.tif]

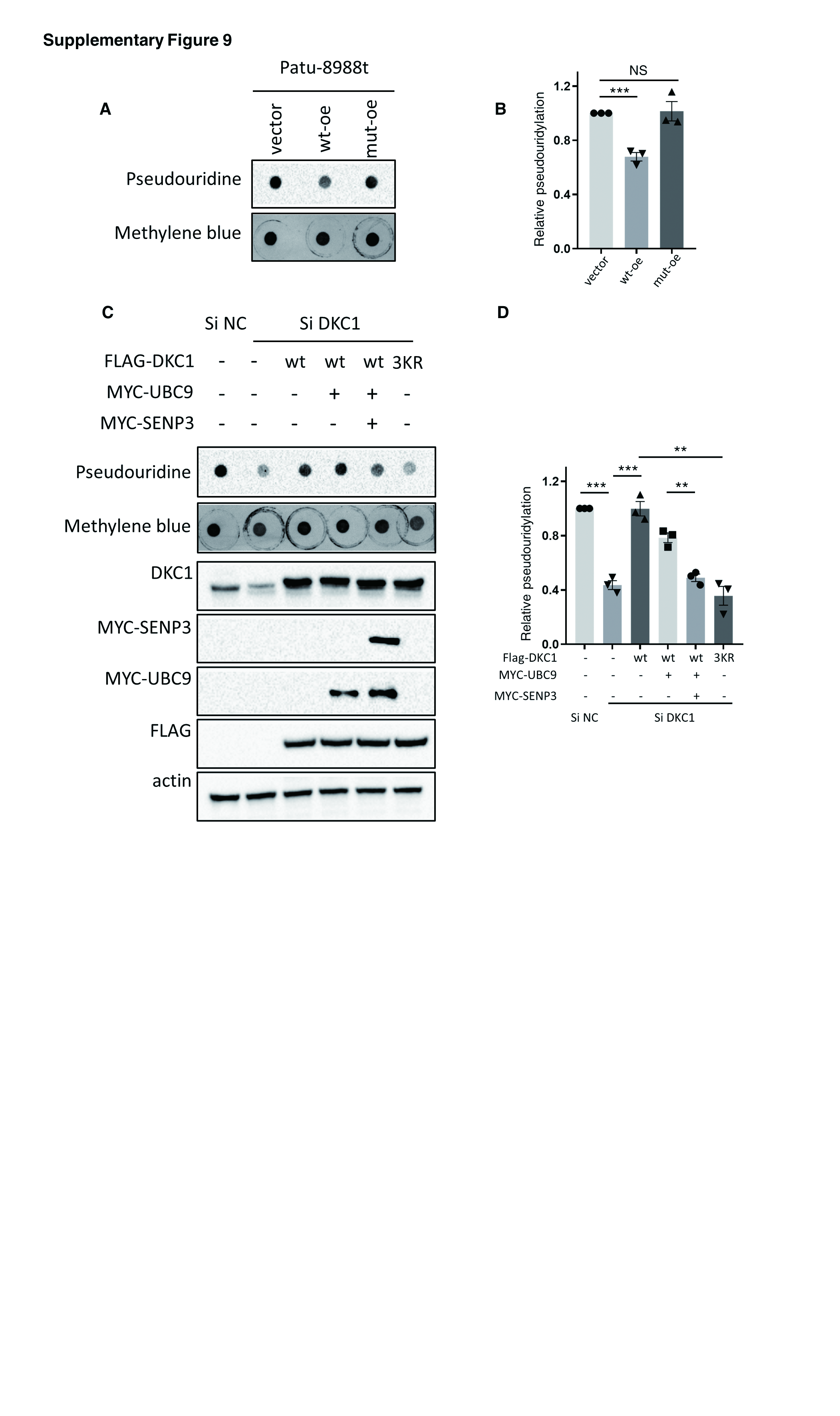

Supplement: Supplementary file 10 — Supplementary Figure 9 [file 41418_2023_1175_MOESM10_ESM.tif]

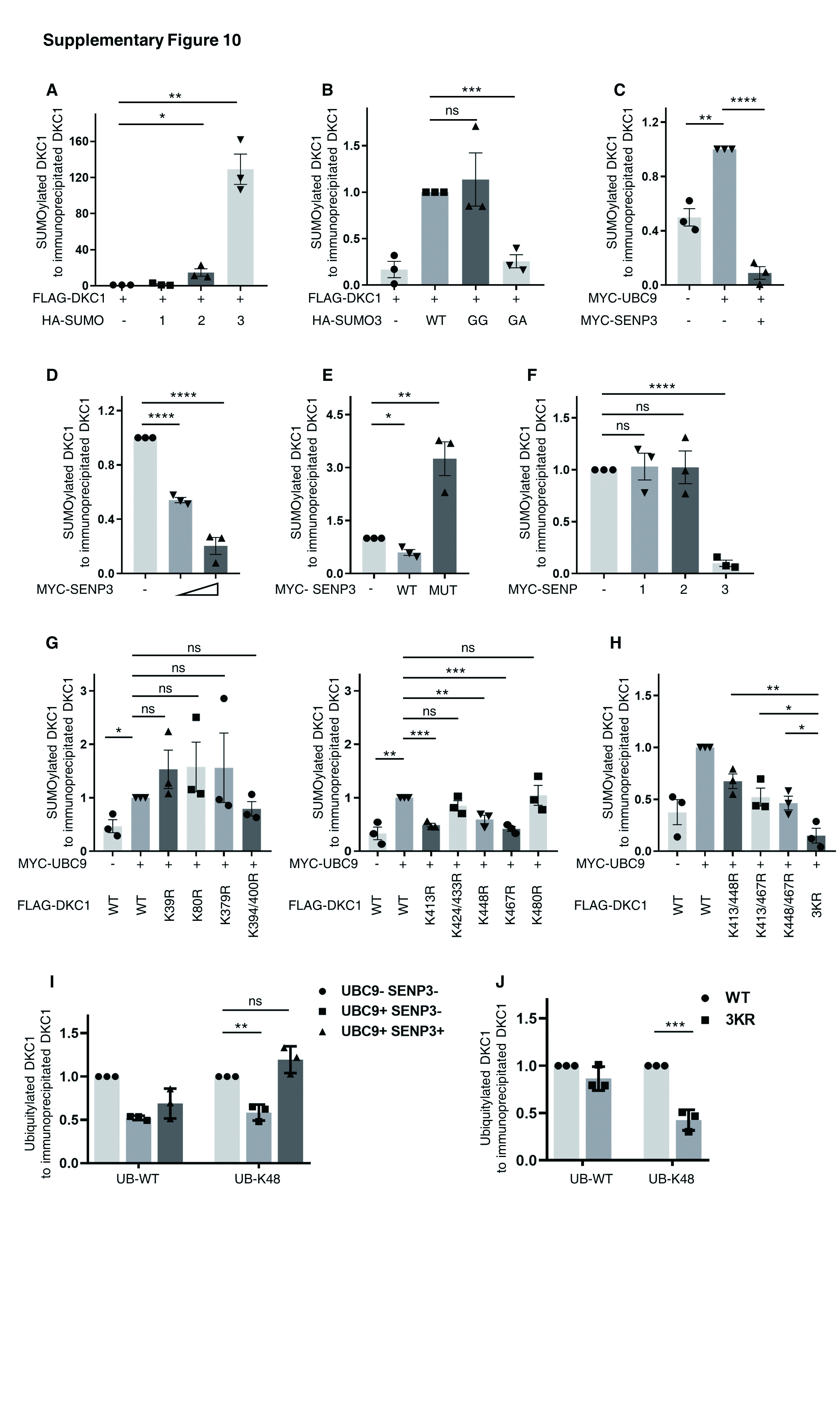

Supplement: Supplementary file 11 — Supplementary Figure 10 [file 41418_2023_1175_MOESM11_ESM.tif]

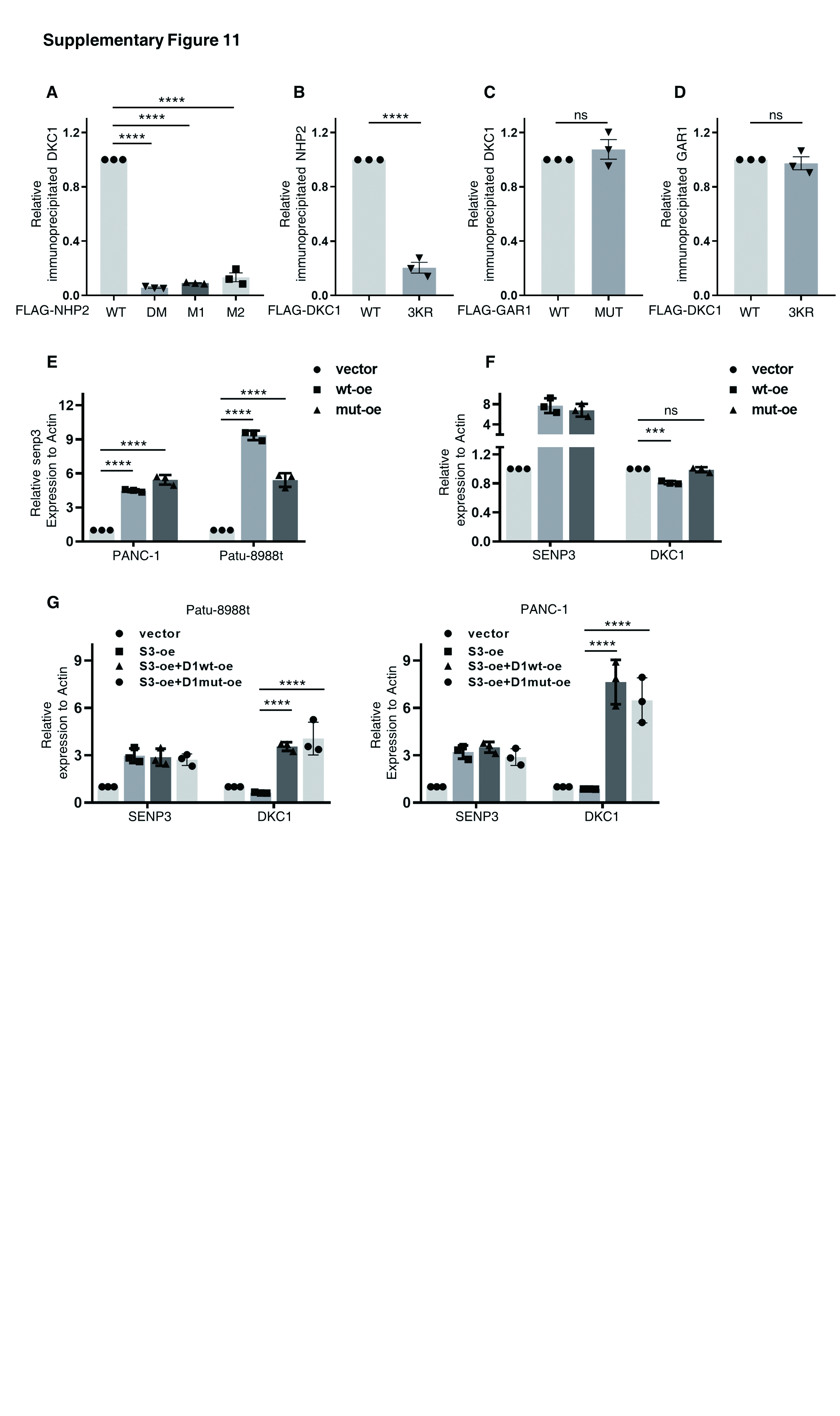

Supplement: Supplementary file 12 — Supplementary Figure 11 [file 41418_2023_1175_MOESM12_ESM.tif]
