# Supplementary material for: SUMO specific peptidase 3 halts pancreatic ductal adenocarcinoma metastasis via deSUMOylating DKC1
Source: Cell Death Differ. 2023 May 15;30(7):1742–56. doi: 10.1038/s41418-023-01175-4 (PMC10307871; doi:10.1038/s41418-023-01175-4)
Supplement: Supplementary file 13 — supplementary information [file 41418_2023_1175_MOESM13_ESM.docx]

Supplementary information for

**SUMO specific peptidase 3 halts pancreatic ductal adenocarcinoma metastasis via deSUMOylating DKC1**

Xiao Wu et al.

This file contains following information:

**Supplementary Figure legends for Fig. S1-11;**

**Supplementary tables 1-7；**

**Supplementary materials and methods.**

**Supplementary Figure Legends**

**Supplementary Figure 1. Liver metastasis preferentially takes place in mice challenged with PDAC cells with low expression of senp3, which is associated with favorable outcomes in patients.**

(A). Knockdown efficiency of sh-RNA lentivirus targeting each SUMO specific peptidase was determined by real-time quantitative PCR.

(B). Primers designed for amplification of specific sequences inserted by pLKO.1 lentivirus infection (up). Validation of the specificity of primer combinations (down).

(C). Sequencing results of inserted genes amplificated from metastatic liver nodules were summarized in this table.

(D). Only senp3 is associated with prognosis of patient suffering from PDAC. The online GEPIA2 database was used to analyze the association of PDAC prognosis with each SUMO specific peptidase.

(E, F). KRAS and TP53 mutation distribution in PDAC patients with different expression of senp3. The cBioPortal web was used to visualize the incidence rate of the indicated mutations in patients stratified by senp3 expression.

(G). Related to Figure 1D, statistical analysis of expression of SENP3 in PDAC and paired nontumor tissues based on the gray value calculated by Image Lab software (Bio-Rad, USA). Paired T test. **p<0.01.

**Supplementary Figure 2. SENP3-mediated anti-metastasis effect depends on DKC1.**

(A). Related to Figure 3C, D, wound healing assay was used to analyze the migration ability of PANC-1 cells infected with indicated overexpression vectors.

(B). PDAC cell lines (Patu-8988t and PDAC) were infected with shRNA lentivirus targeting DKC1 (sh-DKC1), these sh-DKC1cells showed evidently reduced metastasis ability compared with control cell (TRC). However, overexpression of SENP3 (S3-oe) in these sh-DKC1 cell (sh-DKC1 + S3-oe) showed no effect in cell metastasis compared with sh-DKC1 cells. cell metastasis ability was measured with wound healing assay. Statistical analysis was performed according to scratch width at 48h, unpaired t test.

ns, no significance; *p<0.05.

**Supplementary Figure 3.** **Endogenous expression of SENP3 inhibits PDAC cell migration ability.**

(A). SENP3 expression in cells was determined by western blot.

(B). Cell growth curves of Patu-8988t endogenous overexpressing vector or SENP3 were determined with CCK8 assay.

(C). Cell cycle test and apoptosis analysis of indicated cells. unpaired t test.

(D). Transwell assay detected the migration ability of indicated cells. unpaired t test.

(E). Wound healing assay analyzed the migration ability of indicated cells. unpaired t test.

(F). The in vivo migration potency of indicated cells was determined with IVIS imaging.

unpaired t test, ns, no significance, *p<0.05, **p<0.01.

**Supplementary Figure 4. The binding model based on simulated structures of SENP3 and DKC1.**

(A). The predicted models of SENP3 (left) and DKC1 (right).

(B). The binding model of SENP3 and DKC1. The SENP3 is colored in yellow while DKC1 in green. The residues in SENP3 are depicted as yellow sticks while residues in DKC1 are depicted as green sticks. The hydrogen bond and salt bridge interactions between SENP3 and DKC1 are depicted as red and blue dashed lines, respectively.

**Supplementary Figure 5. SENP3 is dispensable for regulating the SUMOylation of other subunits of the H/ACA snoRNP complex, including GAR1, NOP10 and NHP2.**

(A-C). SUMOylation levels of snoRNP proteins, including GAR1(A), NOP10(B) and NHP2(C), were detected in presence of SENP3 or not. HEK293T cells were transfected with a combination of indicated constructs before lysis and pull-down with anti-Flag gels. SUMOylation of indicated molecules was determined by immunoblot assay with anti-HA antibodies after SDS/PAGE resolution in denaturing condition.

(D). Relative SUMOylation level of NHP2 in presence of SENP3 or not was summarized from three independent experiments.

unpaired t test, ns, no significance, ***p<0.001.

**Supplementary Figure 6. Subcellular distribution of DKC1 deletion mutants.**

(A). Illustration for nuclear localization signals in DKC1 sequence.

(B). Cytoplasmic and nuclear extracts of HEK293T cells transfected with indicated Flag-labeled DKC1 deletion constructs were analyzed by immunoblot with antibodies to Flag, α-tubulin (cytoplasmic fraction), and Lamin A/C (nuclear fraction).

(C). localization of DKC1 deletion mutants was determined by fluorescence imaging. Hela cells were transfected with expression vectors encoding GFP-tagged DKC1 mutants, the relative localization was observed by fluorescence microscopy (blue: DAPI, green: DKC1 mutants).

**Supplementary Figure 7. The interaction between DKC1 and NHP2 is disrupted by over-expression of wild-type SENP3.**

(A, B) Endogenous interaction between DKC1 and NHP2 in indicated cells. Antibody against DKC1 was used to pull down endogenous DKC1 in cell lysate, and then NHP2 was detected in the pooled complexes by SDS/PAGE and immunoblot.

**Supplementary Figure 8. DKC1 reverses the anti-metastasis effect of SENP3 and is correlated with SENP3 expression only at the protein level.**

(A). Expression of SENP3 and DKC1 in indicated cells was confirmed by western blot.

(B). Overexpression of wild-type DKC1, but not SUMOylation-resistant DKC1, reversed the decreased migration-ability of PDAC cells mediated by increased SENP3 expression. Statistical analysis was performed according to scratch width at 48h, unpaired t test.

(C). Distribution of patients with different expression of DKC1 in subgroups stratified by SENP3 expression according to IHC scores in PDAC specimens.

(D, E, F). Correlation of senp3 and dkc1 at transcriptional level. Transcriptional expression of senp3 and dkc1 in PDAC tissue was obtained from the datasets GSE15471, GSE16515 and GSE32676. The correlation between these two genes was analyzed using liner-regression test.

ns, no significance; *p<0.05; **p<0.01; ***p<0.001.

**Supplementary Figure 9. SENP3-mediated deSUMOylation of DKC1 decreased cellular pseudouridine levels.**

(A, B). Pseudouridine degrees of indicated cells were detected by dot blots. Unpaired T test.

(C, D). Pseudouridine status of HEK293T cells expressing DKC1 with distinct SUMOylation levels was determined. HEK293T cells were transfected with indicated panel, in which SUMOylation degrees were modulated by UBC9 (up) and SENP3 (down). Also, wildtype and SUMOylation-resistant DKC1(3KR) were resorted into cells, of which endogenous DKC1 was down-regulated with si-RNA targeting 3’UTR of DKC1. Unpaired t test.

Ns, no significance, **p<0.01, ***p<0.001.

**Supplementary Figure 10. The quantification of modified DKC1 for Figure 5-7.**

(A). Relative modification degree of DKC1 by three SUMO modifiers (related to Figure. 5D).

(B). Relative modification degree of DKC1 by different SUMO3 modifiers (related to Figure. 5F).

(C). Relative SUMOylation degree of DKC1 in present with SENP3 or not (related to Figure. 5G).

(D). Relative SUMOylation degree of DKC1 with increasing amount of SENP3 (related to Figure. 5F).

(E). Relative SUMOylation degree of DKC1 with functionally different SENP3 molecules (related to Figure. 5I).

(F). Relative SUMOylation degree of DKC1 with different SENP molecules (related to Figure. 5J).

(G). Relative SUMOylation degree of distinct mutant DKC1 molecules (related to Figure. 6C).

(H). Relative SUMOylation degree of distinct mutant DKC1 molecules (related to Figure. 6D).

(I). Relative ubiquitination degree of DKC1 with distinct SUMOylation status (related to Figure. 7C).

(J). Relative ubiquitination degree of distinct DKC1 molecules (related to Figure. 7D).

The relative modification of DKC1 was calculated by gray values of modified DKC1/ gray values of immunoprecipitated DKC1, and then normalized control groups to 1 and compared the difference between groups via unpaired t test, n=3, ns, no significance, *p<0.05, **p<0.01, ***p<0.001, ****p<0.0001.

**Supplementary Figure 11. The quantification of western blots for Figure 2, 7, S3.**

(A). Relative immunoprecipitated level of DKC1 pulled down by different NHP2 mutants (related to Figure. 7F).

(B). Relative immunoprecipitated level of NHP2 pulled down by different DKC1 mutants (related to Figure. 7G).

(C). Relative immunoprecipitated level of DKC1 pulled down by different GAR1 mutants (related to Figure. 7I).

(D). Relative immunoprecipitated level of GAR1 pulled down by different DKC1 mutants (related to Figure. 7J).

(E). Relative expression of SENP3 to Actin in indicated cell lines (related to Figure. 2B).

(F). Relative expression of SENP3 and DKC1 to Actin in indicated cell lines (related to Figure. 7A).

(G). Relative expression of SENP3 and DKC1 to Actin in indicated cell lines (related to Figure. S3A).

The relative level of target protein (A-D) was calculated by gray values of target molecule/ gray values of immunoprecipitated molecule, and then normalized control groups to 1 and compared the difference between groups via unpaired t test, n=3, ns, no significance, * p<0.05, **p<0.01, ***p<0.001, ****p<0.0001.

**Supplementary tables**

**Supplementary table 1. The clinicopathological characteristics of 70 PDAC patients in the tissue microarray.**

| **Characteristics** | **Values** |
| --- | --- |
| Age, year (mean ± SD) | 63.2 ±10.5 (34-82) |
| Gender (male/female) | 34/36 |
| CA19-9, kU/L (≤37/＞37) | 12/58 |
| TNM stage (I/II/III/IV)  Lymphatic metastasis (Negative, Positive) | 30/18/10/12  42/28 |
| Cancer embolus (Negative, Positive) | 45/25 |
| Vascular invasion (Negative, Positive) | 59/11 |
| Perineural invasion (Negative, Positive) | 50/20 |
| SENP3 IHC score (High/Low) | 35/35 |
| Overall survival, months (mean ± SD) | 15.37 ± 11.74 (2-51) |

| **Variables** | **Overall survival (OS)** | | | | | |
| --- | --- | --- | --- | --- | --- | --- |
|  | HR | | 95%CI | | *P*-value | |
| **Univariate** |  |  |  |  |  |  |
| Age(＞62 or ≤62) | 1.070 | | 0.650 - 1.760 | | 0.7810 | |
| Gender(female/male) | 1.114 | | 0.692 - 1.882 | | 0.5889 | |
| CA19-9(＞37U/L or ≤37U/L) | 2.221 | | 1.247 - 3.953 | | 0.0239 | |
| TNM stage (I+II/III+IV) | 2.774 | | 1.420 - 5.420 | | <0.0001 | |
| Lymphatic metastasis (+/-) | 2.351 | | 1.344 - 4.114 | | 0.0002 | |
| Cancer embolus (+/-) | 1.277 | | 0.742 - 2.197 | | 0.3372 | |
| Vascular invasion (+/-) | 1.968 | | 0.821 - 4.717 | | 0.0379 | |
| Perineural invasion (+/-) | 1.215 | | 0.676 - 2.181 | | 0.4789 | |
| SENP3 protein level(＞4/≤4)^a^ | 0.327 | | 0.188 - 0.568 | | <0.0001 | |
| **Multivariate** |  |  |  |  |  |  |
| SENP3 protein level(＞4/≤4)^a^ | 0.376 | | 0.207-0.682 | | 0.0010 | |
| CA19-9(＞37U/L or ≤37U/L) | 2.289 | | 1.074-4.876 | | 0.0320 | |
| TNM stage (I+II/III+IV) | 1.798 | | 1.347-2.399 | | 0.0004 | |

**Supplementary table 2. Prognostic factors for OS of patients with PDAC determined by univariate and multivariate analysis.**

**CA19-9, carbohydrate antigen 19-9; HR, hazard ratio; CI, confidence interval.**

**a. According to Immunohistochemical (IHC) score, split at median.**

**Supplementary table 3. Correlation between SENP3 and clinical characteristics in PDAC patients.**

| **Characteristics** | **Number of patients** | | **P value** ^a^ |
| --- | --- | --- | --- |
|  | **Low SENP3 expression** | **High SENP3 expression** |  |
| Gender |  |  | 0.6324 |
| Female | 19 | 17 |  |
| Male | 16 | 18 |  |
| Age |  |  | >0.9999 |
| ＞62 | 17 | 17 |  |
| ≤62 | 18 | 18 |  |
| Vascular invasion |  |  | 0.0452^b^ |
| Negative | 26 | 33 |  |
| Positive | 9 | 2 |  |
| Cancer embolus |  |  | 0.2123 |
| Negative | 20 | 25 |  |
| Positive | 15 | 10 |  |
| Perineural invasion |  |  | 0.8798 |
| Negative | 24 | 26 |  |
| Positive | 10 | 10 |  |
| Lymph node invasion |  |  | 0.1432 |
| Negative | 18 | 24 |  |
| Positive | 40 | 12 |  |
| TNM stage |  |  | 0.0065^b^ |
| Ⅰ | 10 | 20 |  |
| Ⅱ | 8 | 10 |  |
| Ⅲ | 6 | 4 |  |
| Ⅳ | 11 | 1 |  |

**a. Chi-square test; b. Fisher's exact test.**

**Supplementary table 4. The primers used for RT-qPCR.**

| genes | Forward primers | Reverse primers |
| --- | --- | --- |
| senp1 | AGTGAACCACAACTCCGTATTC | AAAAGATCGGTCCAAATGTCCTT |
| senp2 | GGCTGGTTAGGATTCTCGGC | GGCAGCATTGTAGAGACTGTTTT |
| senp3 | GGTGGACCAAAAACGTGGAC | ATTCTGCCTGGCCACATTCA |
| senp5 | CTTTAGGTCAGGCCAATGGTC | CAGCAGCCGTAACAAAAGCC |
| senp6 | TCCTGTAAGGTTAAGTCGGCT | AGATAGAGGAGGAGTAGGCTGAT |
| senp7 | GGCCATCTTCATCCGAAATCA | CAAAGGGAGAGTCCAGCGT |
| senp8 | ACTGCGGCAATCAGATGTCTC | GGAACATGGCAATCTCTGCTG |
| dkc1 | GCTAAGTTGGACACGTCTCAG | TGCAAGAGGTGTATAGTGTGTTG |
| gapdh | ACAACTTTGGTATCGTGGAAGG | GCCATCACGCCACAGTTTC |

**Supplementary table 5. Sequences of shRNA targeting indicated genes.**

| genes | sequences | genes | sequences |
| --- | --- | --- | --- |
| senp1 #1 | CCGAAAGACCUCAAGUGGAUU | senp1 #2 | CGAGAAAGAUUGCGCCAGAUU |
| senp2 #1 | ACAAUGCUGCCAGCUUAUUUG | senp2 #2 | CAUGCUGAAACUGGGUAAUAA |
| senp3 #1 | CAUUGGUCCCUCAUCUCUGUU | senp3 #2 | CCUCGCUGACAUUCCACUGGA |
| senp5 #1 | CCUUACCAGAACAUCGUUCUA | senp5 #2 | CCAACACUUGUGCAUUCUGAA |
| senp6 #1 | CACAGGAUUAACAACCAAGAA | senp6 #2 | GACAGAACUAACAGAAGAGAA |
| senp7 #1 | GUCAUCUCUCUAGACCAUAAA | senp7 #2 | CCCGUUCAGAAGUUGAUUGUU |
| senp8 #1 | GCUGGCUCAAUGACCAUAUUA | senp8 #2 | CCCUGCAUACAUCACAAAGAA |
| dkc1 | UAUGUUGACUACAGUGAGUCU |  |  |

**Supplementary table 6. Antibodies used in this paper.**

| **Antibodies** | **Sources** | **Identifiers** | **Application** |
| --- | --- | --- | --- |
| SENP3 | Immunoway | Cat# YT4238 | IHC |
| SENP3 | Cell Signaling Technology | Cat# 5591 | WB, IP |
| DKC1 | abcam | Cat# 156877 | IHC |
| DKC1 | Santa Cruz | Cat# sc-373956 | WB, IP |
| HA-tag | Cell Signaling Technology | Cat# 3724 | WB |
| Myc-tag | Cell Signaling Technology | Cat# 2276 | WB |
| ACTIN | Cell Signaling Technology | Cat# 3700 | WB |
| Flag-tag | Sigma Aldrich | Cat# 1804 | WB |

**Supplementary table 7. The contact list between SENP3 and DKC1 (related to Fig. S4)**

| **Chain 1** | **Residue** | **Chain 2** | **Residue** | **Interaction type** |
| --- | --- | --- | --- | --- |
| SENP3 | Glu3: OE2 | DKC1 | Lys471: NZ | Salt bridge |
| SENP3 | Met135: SD | DKC1 | Lys379: NZ | Hydrogen bond |
| SENP3 | Arg158: NH2 | DKC1 | Asp491: OD2 | Salt bridge |
| SENP3 | Arg158: NH1 | DKC1 | Asp491: OD1 | Hydrogen bond |
| SENP3 | Arg158: NH2 | DKC1 | Lys498: O | Hydrogen bond |
| SENP3 | Arg159: O | DKC1 | Lys502: CD | Hydrogen bond |
| SENP3 | Lys165: CD | DKC1 | Asp495: OD1 | Hydrogen bond |
| SENP3 | Thr229: OG1 | DKC1 | Asp495: OD1 | Hydrogen bond |
| SENP3 | Cys243: SG | DKC1 | Gly75: O | Hydrogen bond |
| SENP3 | Cys243: SG | DKC1 | Ser76: CA | Hydrogen bond |
| SENP3 | Cys243: O | DKC1 | Asn77: ND2 | Hydrogen bond |
| SENP3 | Cys243: SG | DKC1 | Lys339: CD | Hydrogen bond |
| SENP3 | Asn522: OD1 | DKC1 | Lys394: NZ | Hydrogen bond |

**Supplementary materials and methods.**

**Cell culture.**

The human PDAC cell lines (Patu-8988t and PANC-1), HEK293T cells as well as Hela cells were purchased from the Cellcook Co., Ltd (Guangzhou, China) and cultured in Dulbecco's Modified Eagle Medium (DMEM, Gibco) supplemented with 10% heat-inactivated fetal bovine serum (FBS, Gibco), 100 U/ml penicillin .and 100 mg/ml streptomycin (Gibco) in a humidified atmosphere containing 5% CO_2_ at 37°C.

**Tissue microarray and immunohistochemical staining**

Human PDAC tissue microarrays (ZL-PACSur1602) were purchased from Zhuolibiotech Company Co., Ltd. (Shanghai, China), clinical and pathological information of the samples was provided by the assay manufacturer and illustrated in supplementary tables 1-3. IHC staining of paraffin-embedded tissues with antibodies against SENP3 and DKC1 was performed according to the previously described standard procedures. The expression level of molecules was scored semi-quantitatively based on both the intensity of staining and distribution using the immunoreactive score (IRS). Staining intensity (SI) was recorded as 0, 1, 2, 3, referring to negative, weak, moderate and strong staining respectively. The percentage of positive cells (PP) was assigned as 0=0%, 1=1-25%, 2=25-50%, 3=50-75%, 4=75-100%. Briefly, IRS = SI x PP (ranging from 1 to 12), and the high expression referred to IRS more than 4.

**Real-time quantitative PCR.**

Total RNA was extracted from cells using TRIzol reagent (Invitrogen, USA) and then reversed transcribed with HiScript Q Select RT SuperMix (Vazyme, China) to synthesize cDNA. Quantitative real-time PCR was performed with SYBR Green Mix (Vazyme, China) and indicated primers (attached in supplementary table 4) were used to assess transcript level of each gene.

**Mass spectrum.**

To seek the potential substrates of SENP3, immunoprecipitated complexes pulled down with anti-SENP3 antibodies were performed as described in immunoprecipitation methods. Preparation of digested peptides, chromatography separation with Easy nLC 1200 system (ThermoFisher, USA) as well as subsequent data acquisition on the Q Exactive mass spectrometer (ThermoFisher, USA) were performed by staff of Wininnovate Bio Company Co., Ltd (Shenzhen, China).

**Transwell invasion assay.**

As previously described, 6×10^4^ cells suspended in 200μL serum-free medium were seeded in the upper chamber coated with Matrigel (Corning, cat# 354234). 24 hours later, invasive cells on the lower surface were fixed and then stained using 0.5% crystal violet for 15 min. The number of invaded cells was imaged and counted using a light microscope.

**Wound healing assay.**

Cells were seeded in 6-well plates and cultured in the incubator until cell confluence reached around 90%. Scratches were made on the cell monolayer with a pipette tip and then washed twice with PBS to remove cell debris. Scratch images were taken at 0h, 24h and 48h after the scratch with a light microscope.

**CCK8 assay.**

Cell viability was measured at 0, 24, 48,72, 96h for each cell line with Cell Counting Kit-8 (CCK8) (Dojindo, Japan) according to the manufacturer’s instructions. Absorbance values were measured at the wavelength of 450nm as a representation of cell viability.

**Cell cycle assay.**

The PDAC cells are infected with the indicated lentivirus. Cells were collected and then subjected to cell cycle assay using the cell cycle staining kit (BestBio, China). The data was collected by flow cytometry and analyzed with CytExpert software (Beckman Coulter, USA).

**Apoptosis assay.**

Percentages of apoptotic cells in infected cells were assessed using the Annexin V-FITC/PI Apoptosis Kit (BestBio, China) according to the manufacturer's instructions. The data was collected by flow cytometry and analyzed with CytExpert software (Beckman Coulter, USA).

**Subcellular fractionation:**

Nuclear and cytoplasmic protein extraction Kit (Beyotime) was used to extract nuclear and cytoplasmic protein of HEK293T cells transfected with indicated plasmids.

**Dot blot:**

To detect the Pseudouridine degree of indicated cell, total RNA was extracted using TRIzol reagent (Invitrogen) followed by quantification with NanoDrop Spectrophotometer (thermoScientific). After boiling for 5 min at 95℃ and then chilling on ice immediately, an equal amount of total RNA was loaded onto nylon membrane. After crosslinking with UV, the membrane was visualized using antibody against pseudouridine (MBL, #D347-3). After chemiluminescence detection, the membrane was then stained with methylene blue as a loading control.

**Molecular docking**

HDOCK sever11 was used for molecular docking simulation of SENP3 with DKC1. The 3D structures of SENP3 and DKC1 predicted by I-TASSER12, an online resource for automated protein structure prediction and structrure-based function annotation, were used in the docking simulation. SENP3 is set as receptor while DKC1 as ligand. The HDOCK server automatically predicts their interaction through a hybrid algorithm of template-based and template-free docking. The predicted complex structures were provided for further evaluation. Molecular graphics were generated by PyMOL.
