# Supplementary material for: SUMO specific peptidase 3 halts pancreatic ductal adenocarcinoma metastasis via deSUMOylating DKC1
Source: Cell Death Differ. 2023 May 15;30(7):1742–56. doi: 10.1038/s41418-023-01175-4 (PMC10307871; doi:10.1038/s41418-023-01175-4)

Uncropped original western blots

Fig.1D

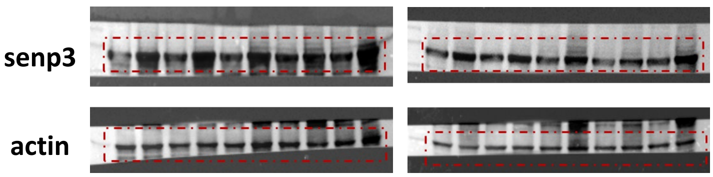

Fig.2B

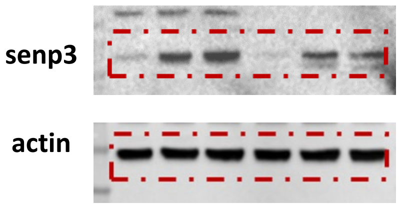

Fig.4C

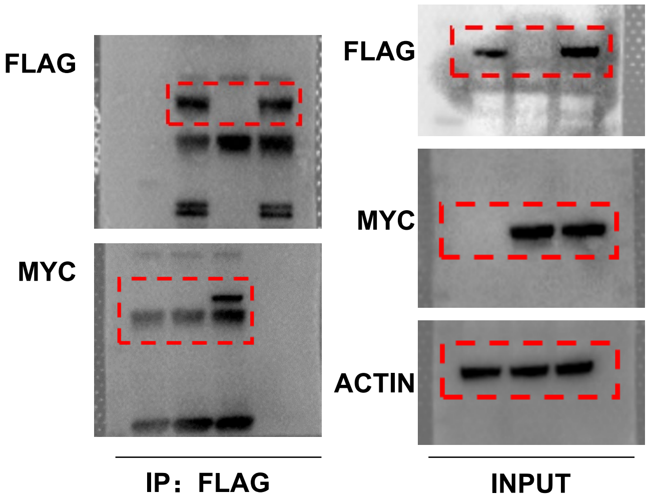

Fig.4D

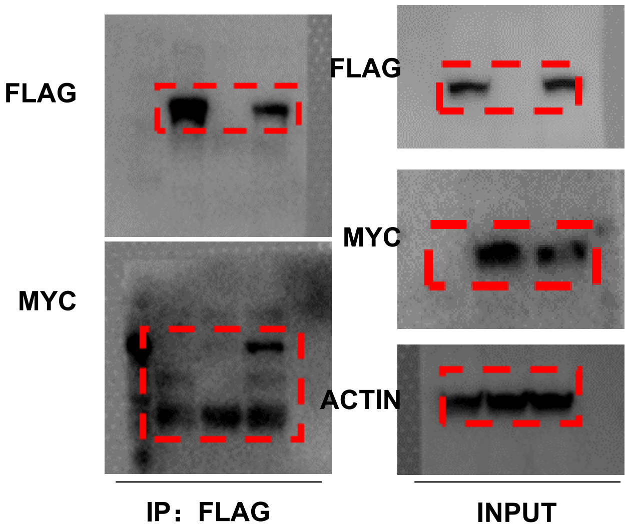

Fig.4E

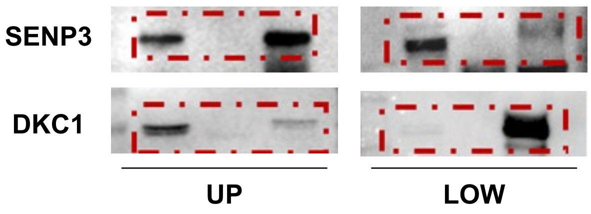

Fig.4G

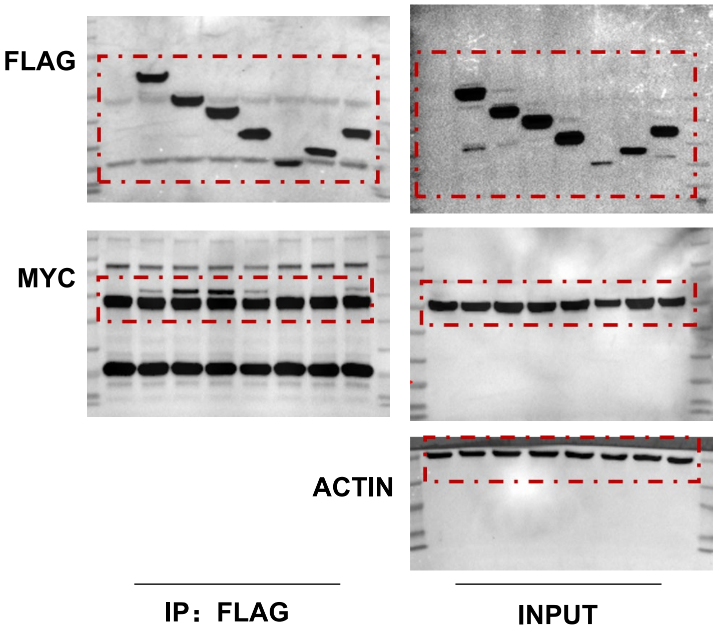

Fig. 4I

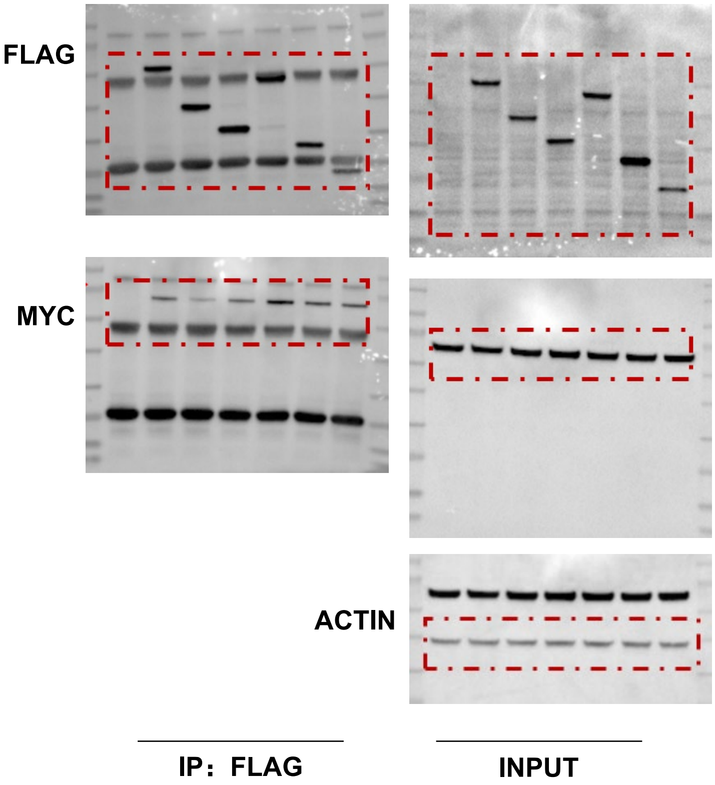

Fig. 4J

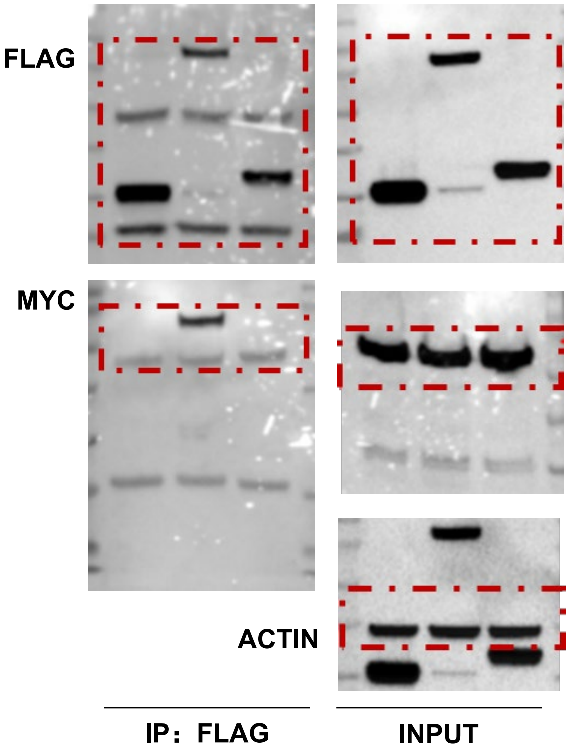

Fig. 5A

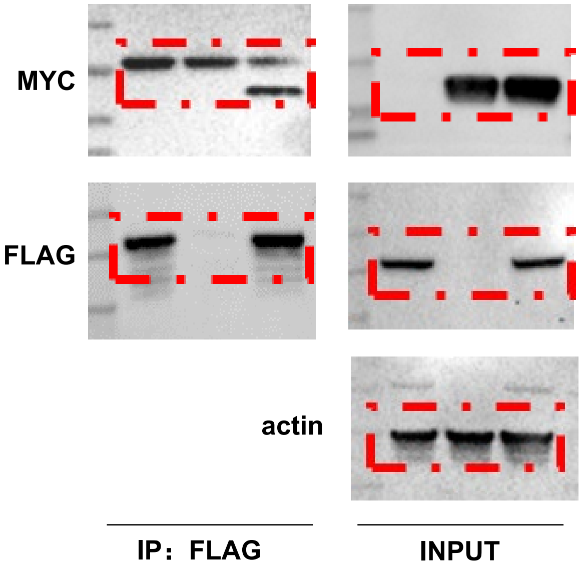

Fig. 5B

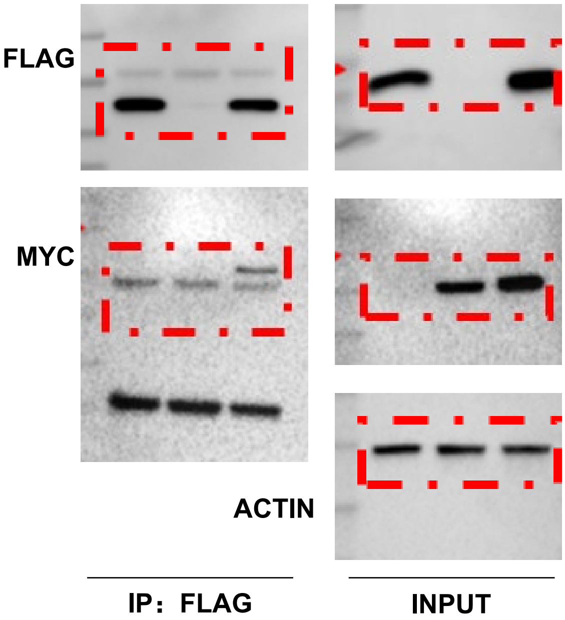

Fig. 5C

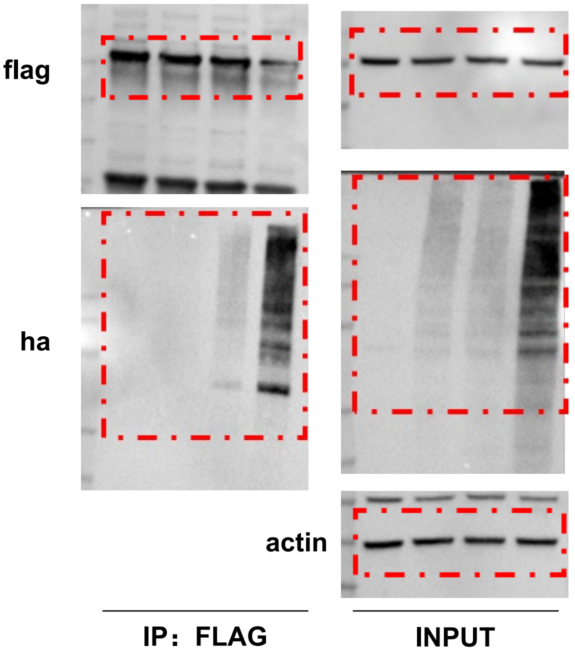

Fig. 5D

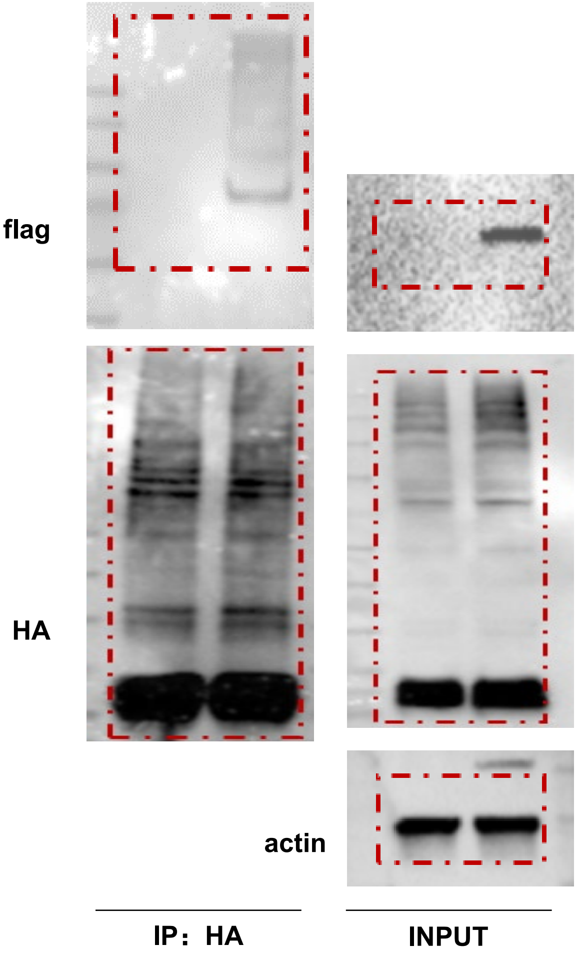

Fig. 5G

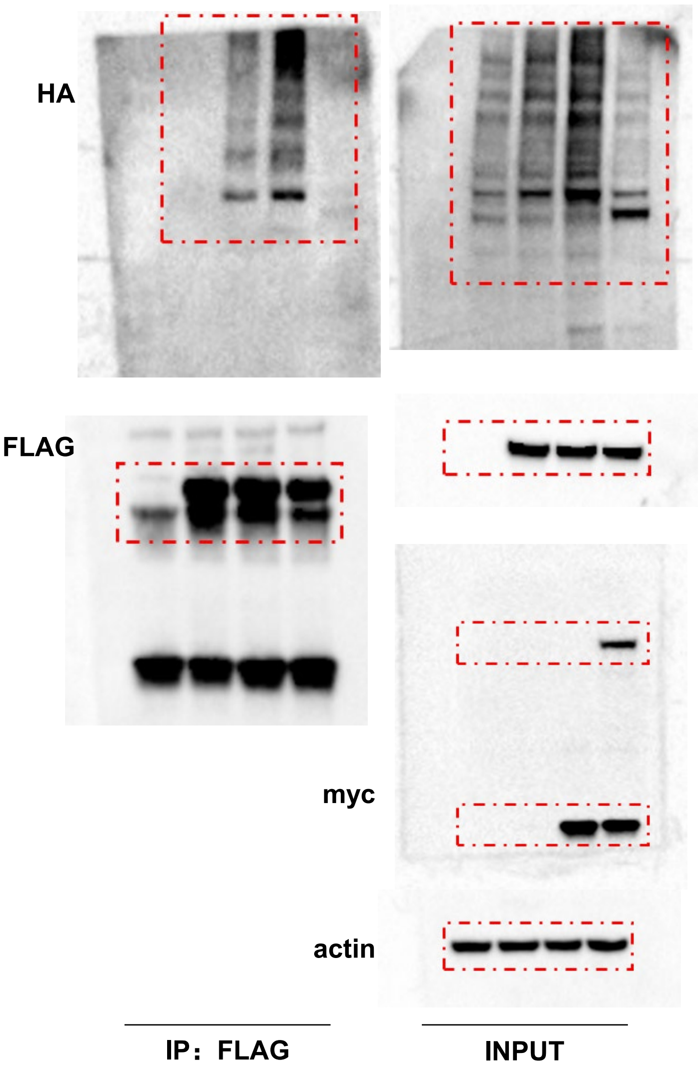

Fig. 5E

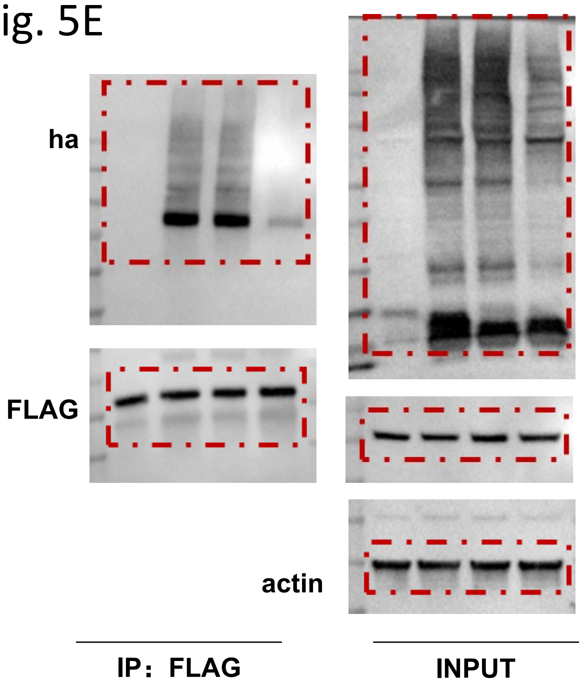

Fig. 5H

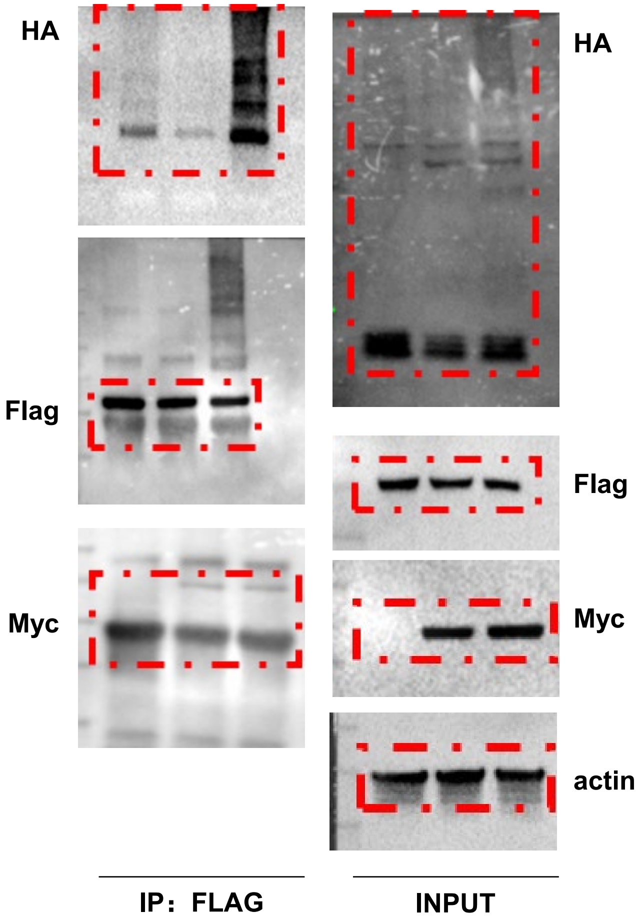

Fig. 5I

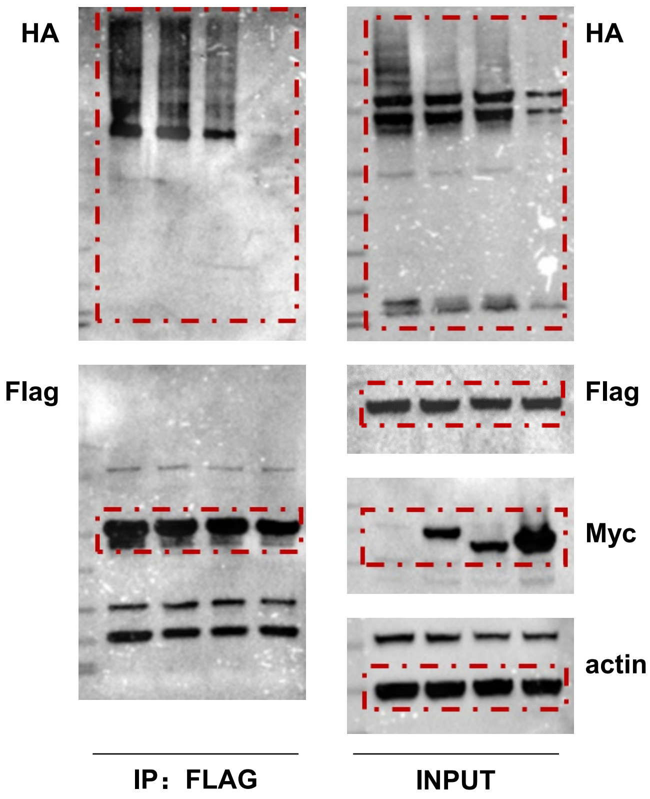

Fig.5J

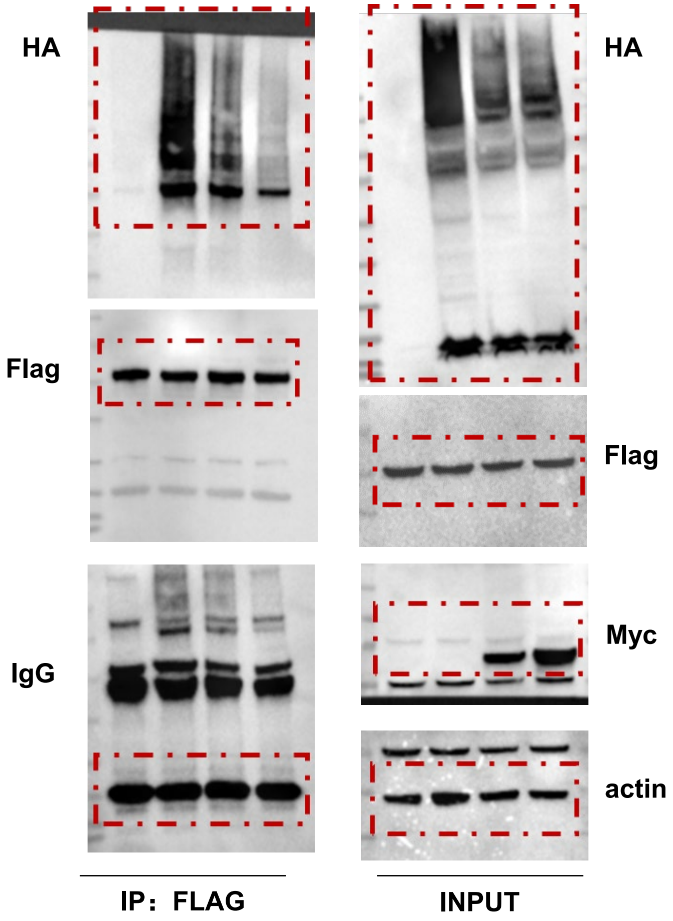

Fig. 6B

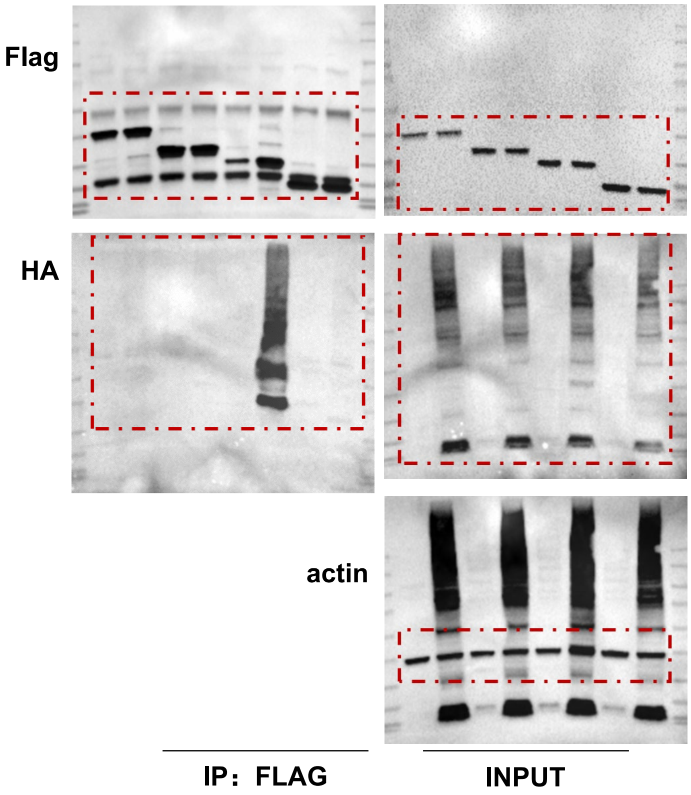
Flag
HA
actin
IP：FLAG
INPUT

Fig. 6C

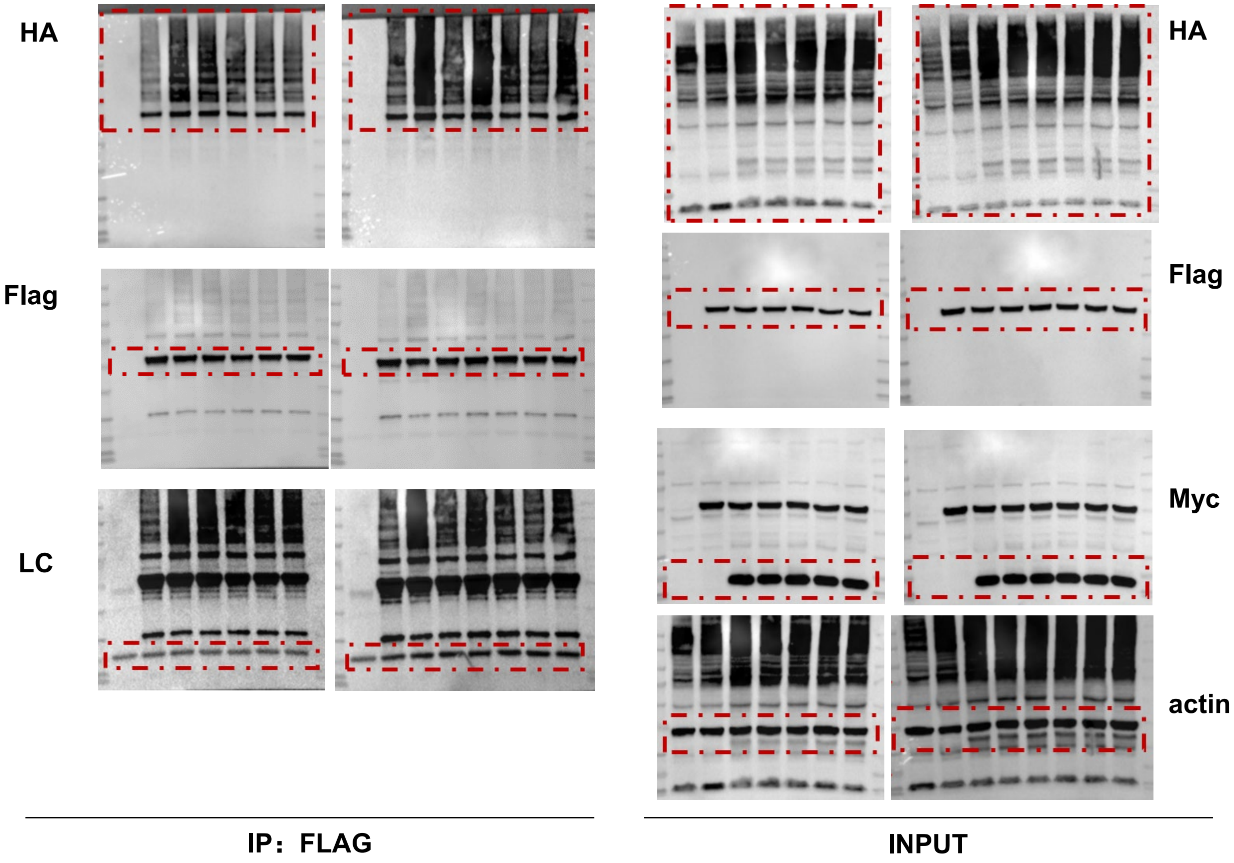
HA
Flag
LC
HA
Flag
Myc
actin
IP：FLAG
INPUT

Fig. 6D

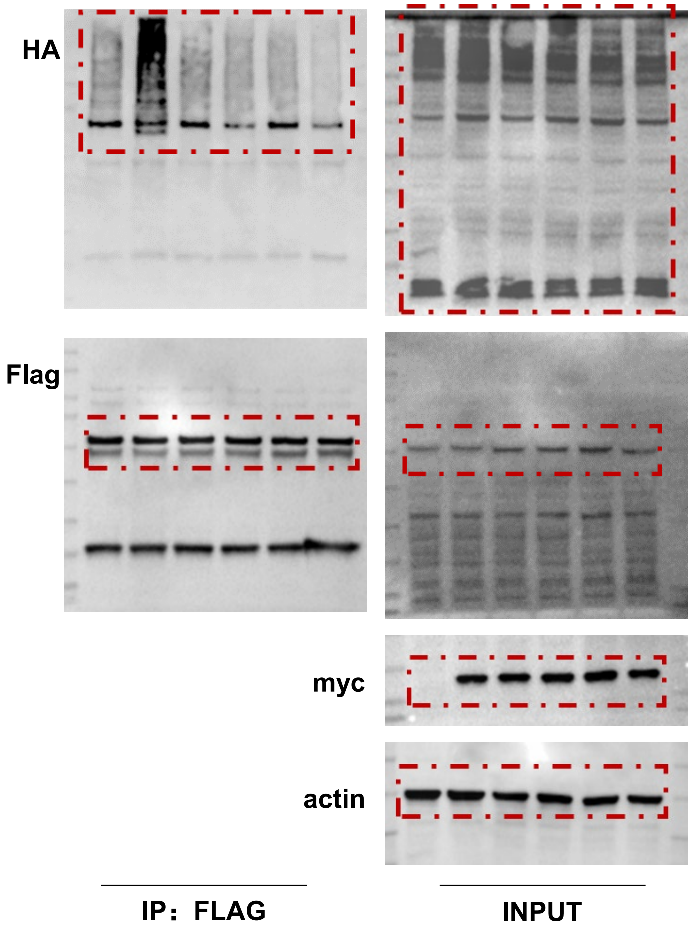

Fig.7A

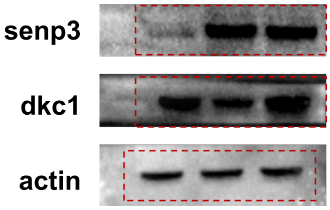

Fig.7F

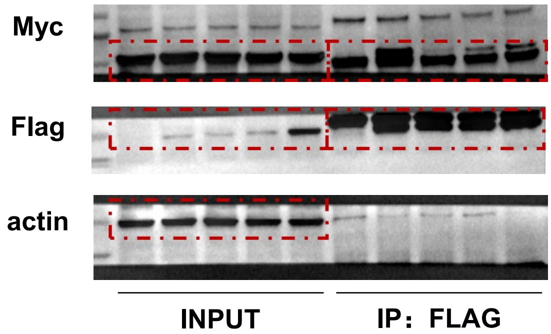

Fig.7C

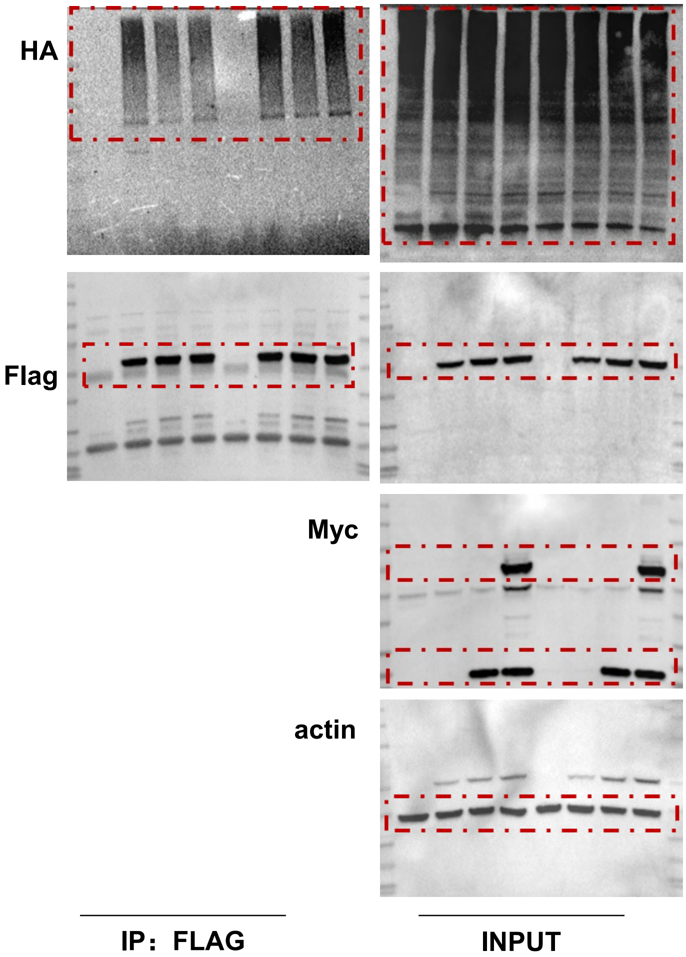

Fig.7D

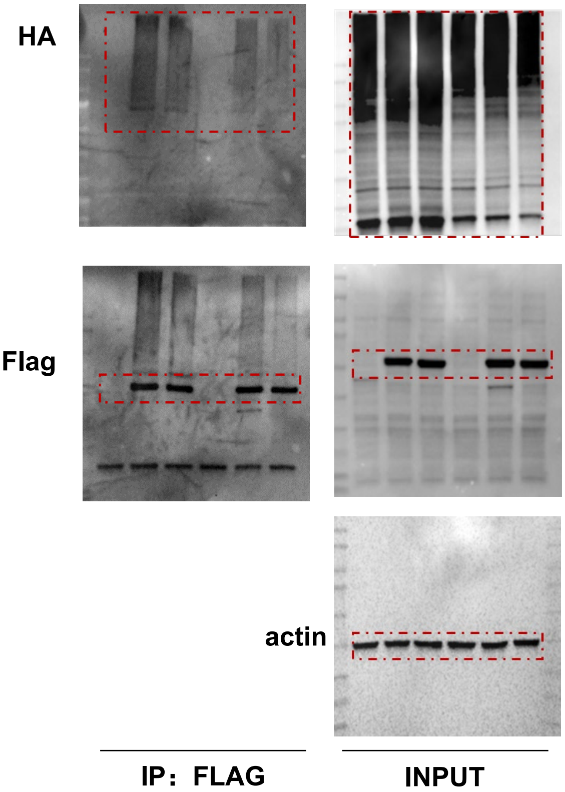

Fig.7G

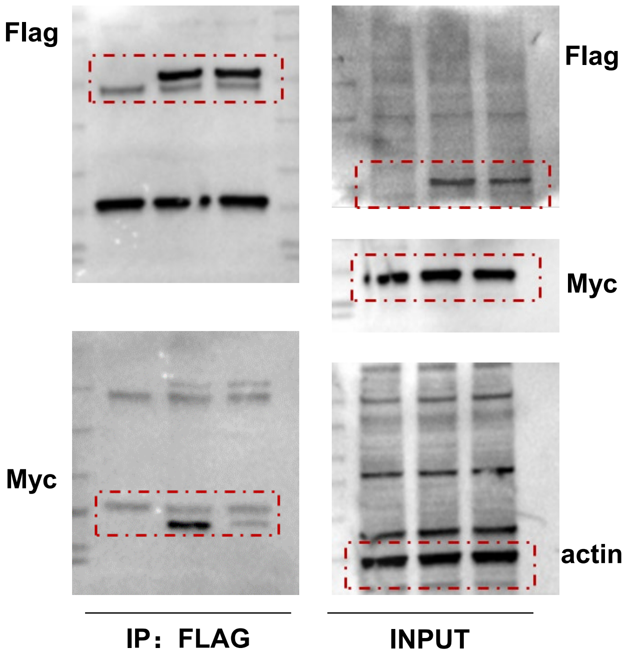

Fig. 7I

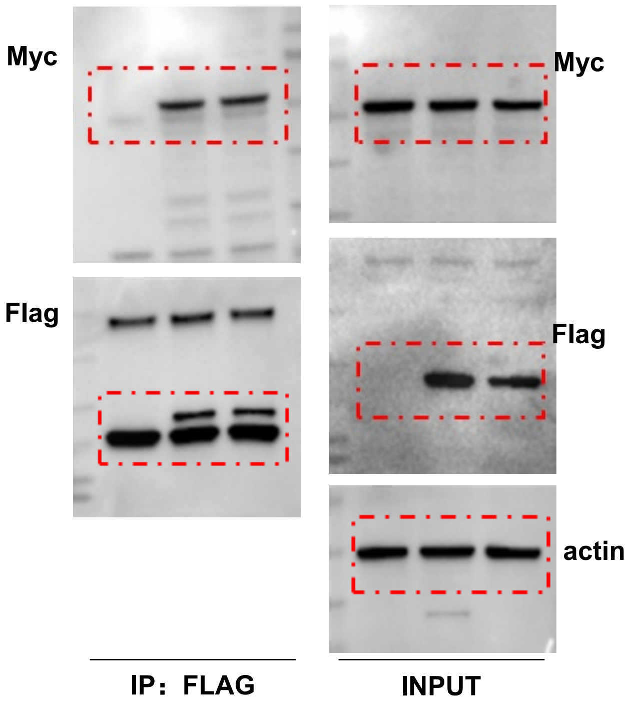

Fig. 7J

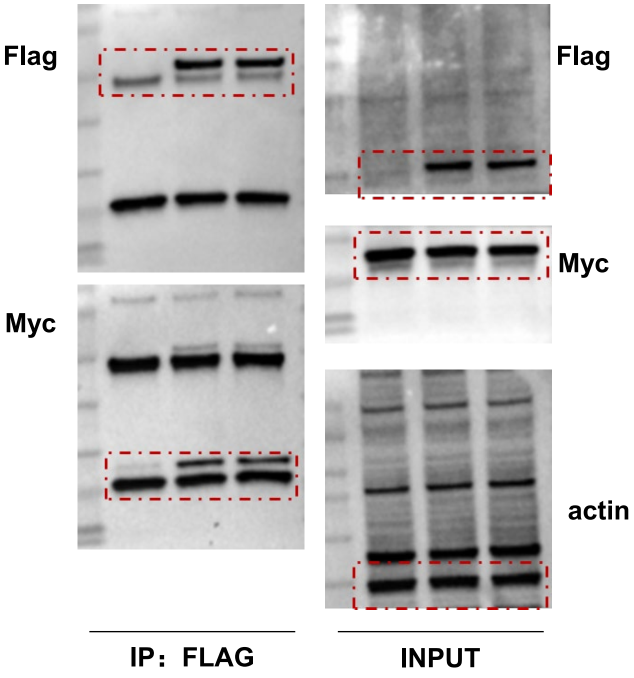

Fig.S3A

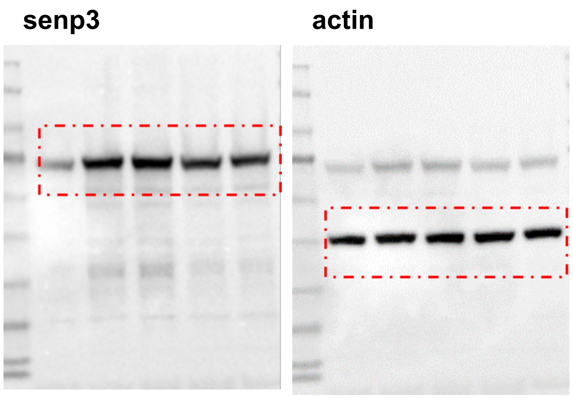

Fig.S5A

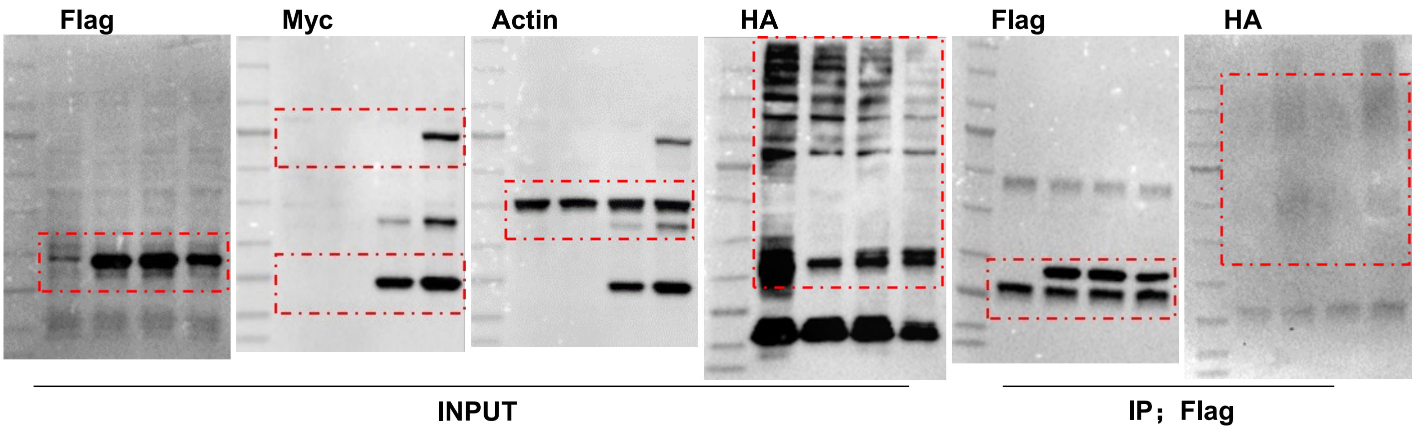

Fig.S5B

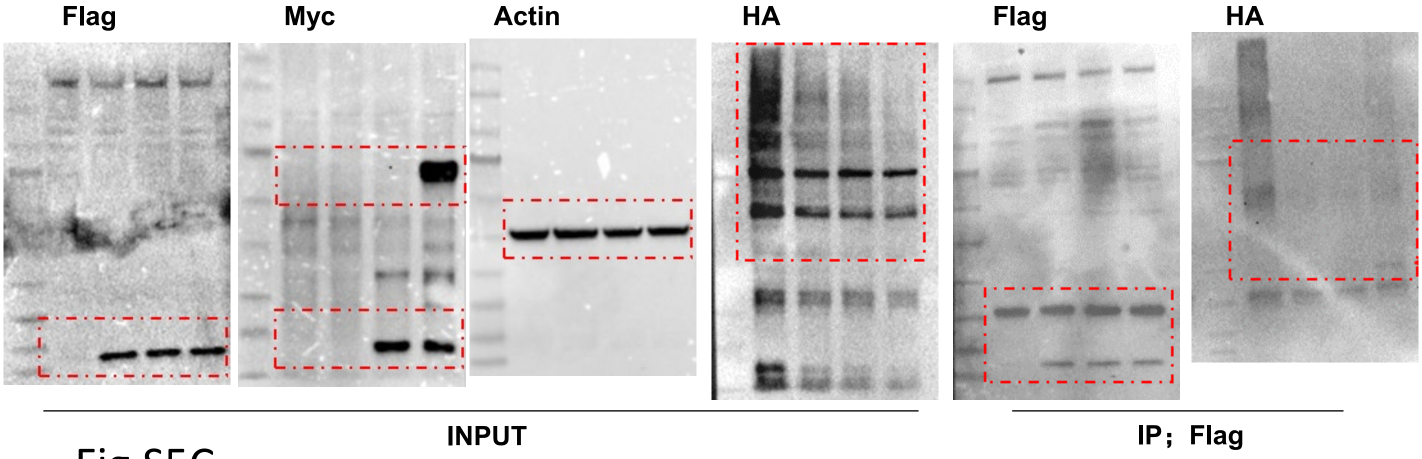

Fig.S5C

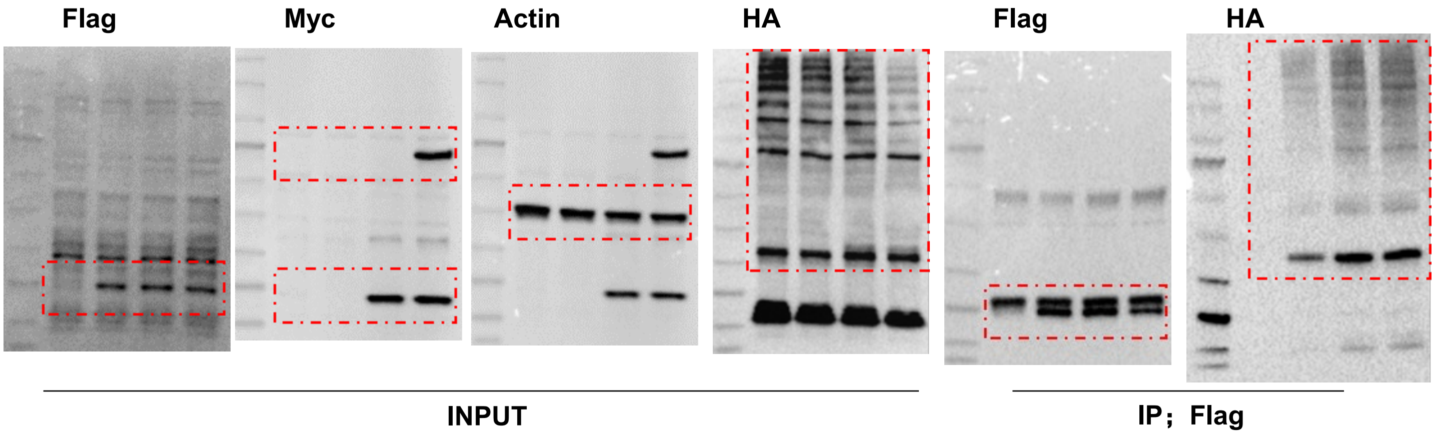

Fig.S6B

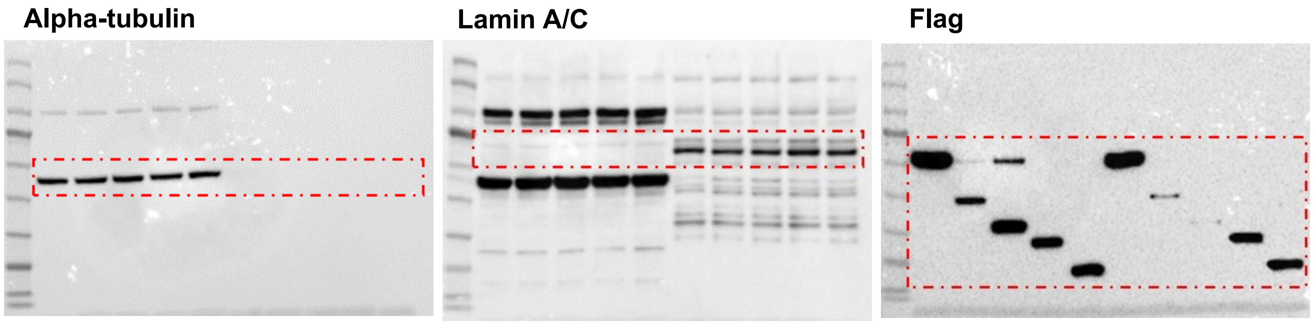

Fig.S7A

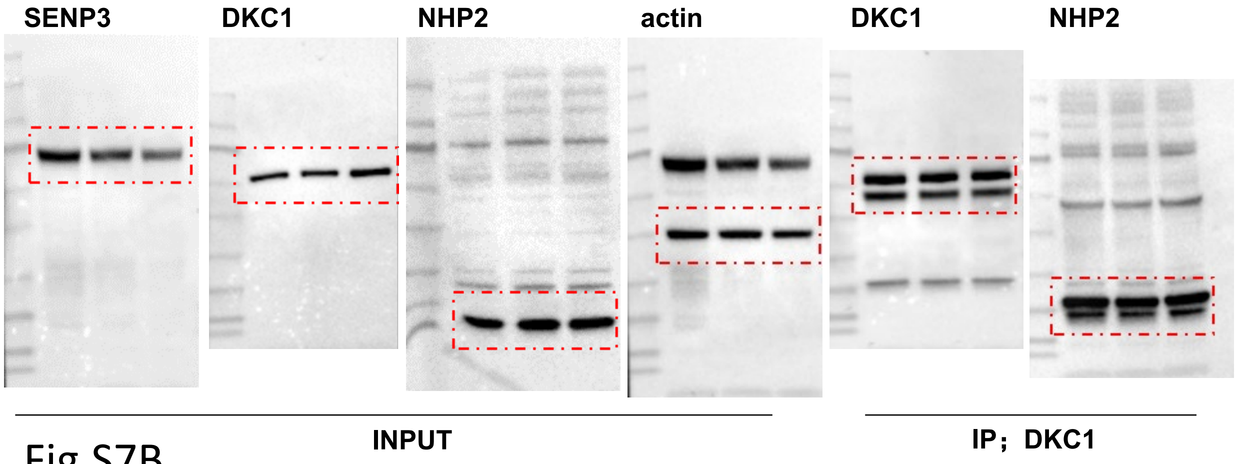

Fig.S7B

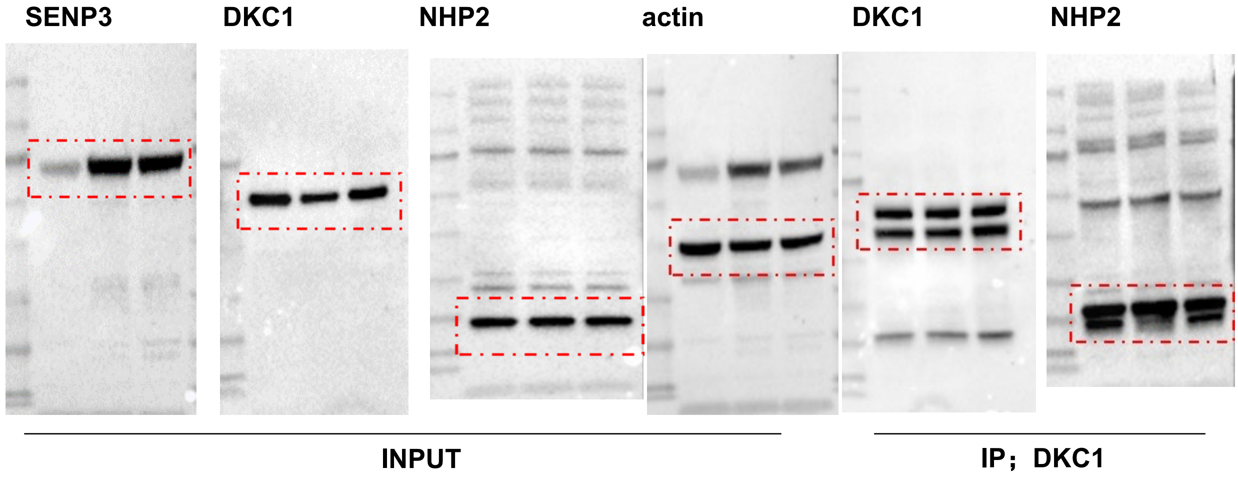

Fig. S8A

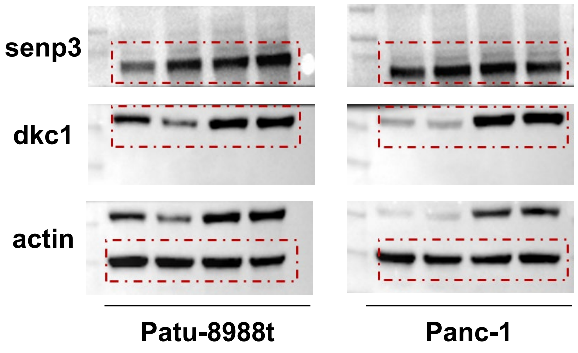

Fig.S9A

Pseudouridine **Methylene blue**

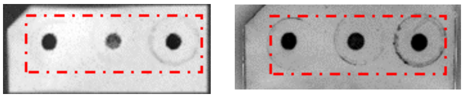

Fig.S9C

DKC1 **Flag** **actin** **myc**

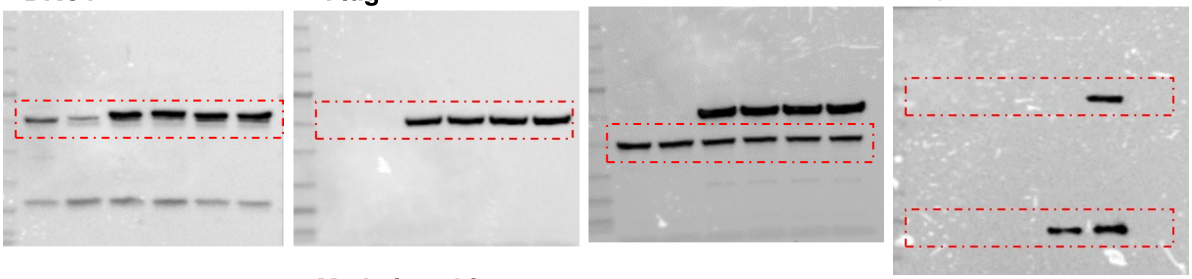

Pseudouridine **Methylene blue**

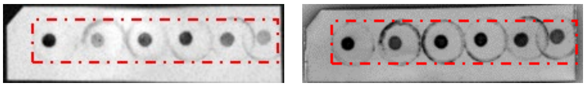

Supplement: Supplementary file 14 — uncropped original western blots [file 41418_2023_1175_MOESM14_ESM.pdf]
